# The Pseudouridine Synthase RPUSD4 Is an Essential Component of Mitochondrial RNA Granules*[Fn FN2]

**DOI:** 10.1074/jbc.M116.771105

**Published:** 2017-01-12

**Authors:** Sofia Zaganelli, Pedro Rebelo-Guiomar, Kinsey Maundrell, Agata Rozanska, Sandra Pierredon, Christopher A. Powell, Alexis A. Jourdain, Nicolas Hulo, Robert N. Lightowlers, Zofia M. Chrzanowska-Lightowlers, Michal Minczuk, Jean-Claude Martinou

**Affiliations:** From the ‡ Department of Cell Biology, University of Geneva, 30 quai Ernest-Ansermet, 1211 Genève 4, Switzerland,; § Medical Research Council Mitochondrial Biology Unit, Hills Road, Cambridge CB2 0XY, United Kingdom,; ¶ Graduate Program in Areas of Basic and Applied Biology (GABBA), University of Porto, Porto 4200-135, Portugal,; ‖ Wellcome Trust Centre for Mitochondrial Research, Institute of Cell and Molecular Biosciences, The Medical School, Newcastle University, Framlington Place, Newcastle upon Tyne, NE2 4HH, United Kingdom, and; ** Institute of Genetics and Genomics of Geneva, Université de Genève, 1211 Genève 4, Switzerland

**Keywords:** mitochondria, ribosome, RNA, RNA-binding protein, RNA modification, mitoribosome biogenesis, OXPHOS, pseudouridine, RPUSD

## Abstract

Mitochondrial gene expression is a fundamental process that is largely dependent on nuclear-encoded proteins. Several steps of mitochondrial RNA processing and maturation, including RNA post-transcriptional modification, appear to be spatially organized into distinct foci, which we have previously termed mitochondrial RNA granules (MRGs). Although an increasing number of proteins have been localized to MRGs, a comprehensive analysis of the proteome of these structures is still lacking. Here, we have applied a microscopy-based approach that has allowed us to identify novel components of the MRG proteome. Among these, we have focused our attention on RPUSD4, an uncharacterized mitochondrial putative pseudouridine synthase. We show that RPUSD4 depletion leads to a severe reduction of the steady-state level of the 16S mitochondrial (mt) rRNA with defects in the biogenesis of the mitoribosome large subunit and consequently in mitochondrial translation. We report that RPUSD4 binds 16S mt-rRNA, mt-tRNA^Met^, and mt-tRNA^Phe^, and we demonstrate that it is responsible for pseudouridylation of the latter. These data provide new insights into the relevance of RNA pseudouridylation in mitochondrial gene expression.

## Introduction

Gene expression in mitochondria is a unique and complex process that requires tightly coordinated expression of the mitochondrial and nuclear genomes to allow the biogenesis of the electron transport chain and of the ATP synthase. This oxidative phosphorylation (OXPHOS)^3^ system is the main producer of ATP in cells and in humans is composed of around 100 proteins of which 13 of the most hydrophobic components are encoded by the mitochondrial DNA (mtDNA). In addition, the minimal mitochondrial genome encodes 22 mt-tRNAs and two mt-rRNAs (12S and 16S) essential for protein synthesis in mitochondria. All the other proteins involved in mtRNA synthesis and processing, translation, and post-translational modification of the mitochondrial gene products are encoded in the nucleus and imported from the cytosol to exert their functions (1). Mutations in many of the genes encoding these proteins (2), as well as mutations in the mtDNA, have been associated with a highly heterogeneous group of severe human pathologies for which the search for efficient treatment strategies remains a major challenge (3).

Both strands of the mtDNA are transcribed in the form of long polycistronic precursor RNAs, which are then processed to release the individual mt-tRNAs, mt-rRNAs, and mt-mRNAs (4, 5). These different RNA species are variously modified during or after transcription by, for example, polyadenylation, methylation, pseudouridylation, and, in the case of tRNAs, CCA addition and aminoacylation. Although less well characterized than other types of RNA modifications, pseudouridine (Ψ or 5-ribosyluracil) was the first to be discovered and is the most abundant RNA modification described thus far. It is generated through uridine-specific isomerization, resulting in the formation of an extra hydrogen bond donor at its non-Watson-Crick edge (6). As a consequence, the presence of pseudouridine generally rigidifies RNA secondary structures; thus it has been proposed to contribute toward the stabilization of a particular structural motif. Accordingly, pseudouridylation is found predominantly in tRNAs, rRNAs, and other small non-coding RNAs (7) for which proper structure is critical for function. In humans, 13 different pseudouridine synthases have been identified. Of these 13 enzymes, five have been shown (PUS1) or predicted (PUSL1, TRUB2, RPUSD3, and RPUSD4) to localize to mitochondria (8, 9). Pseudouridine has been found at several highly conserved sites in bovine mt-tRNAs (10) as well as at position 1397 of the human 16S mt-rRNA (11, 12). To date, more is known about the general role of pseudouridylation in mitochondria than about the enzymes responsible for it. The only exception in humans is PUS1, which is responsible for the formation of Ψ27 and Ψ28 in a subset of mt-tRNAs with loss of function mutations of this enzyme having been identified in patients presenting myopathy, lactic acidosis, and sideroblastic anemia (13). The four remaining mitochondrial pseudouridine synthases are still uncharacterized, and their pseudouridylation target sites have yet to be assigned.

The various stages of mt-RNA processing in mitochondria appear to be spatially organized within foci, named mitochondrial RNA granules (MRGs). The existence of mitochondrial foci containing newly synthesized RNA was first reported by Iborra *et al.* (14). However, little attention was given to these compartments until more recently when some groups, including ours, reported that a number of proteins involved in mtRNA processing, mitoribosome subunit assembly, and translation-associated factors was also found to localize in these structures (for a review, see Ref. 15). The identification of such a panel of MRG-associated proteins led us to conclude that many, if not all, stages of mitochondrial gene expression are centered on these granules. However, due to the technical challenges inherent in the purification of intact MRGs, a complete description of their proteome is yet to be established.

Thanks to the small scale of mitochondrial proteome, here we have expressed several potential mitochondrial RNA-binding proteins and identified novel MRG components using a microscopy-based approach. Among these, we have chosen to focus our attention on the hitherto uncharacterized putative mitochondrial pseudouridine synthase, RPUSD4. We show that *RPUSD4* is an essential gene in human cells and that its silencing leads to a severe defect in mitochondrial respiratory activity. More specifically, we demonstrate that down-regulation of this enzyme causes a decrease of the 16S mt-rRNA, resulting in a defective biogenesis of the large mitochondrial ribosomal subunit (mt-LSU) and, as a consequence, a severe reduction of mitochondrial protein synthesis. Finally, we report that RPUSD4 interacts physically with the 16S mt-rRNA, mt-tRNA^Met^, and mt-tRNA^Phe^, and we present evidence that indicates that RPUSD4 is responsible for the formation of pseudouridine in the mt-tRNA^Phe^.

## Results

**FIGURE 1.**
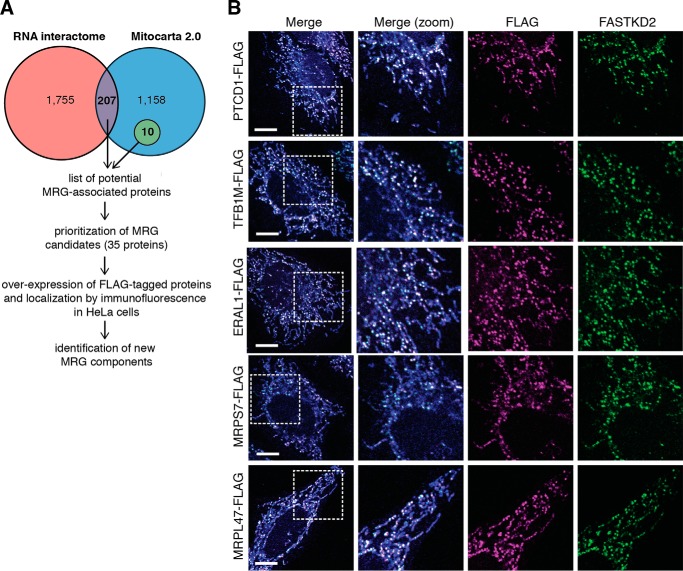
**Identification of RNA-binding proteins associated with MRGs.** *A*, schematic representation of the approach used to identify new MRG components. *B*, representative confocal images of different classes of MRG-associated proteins. HeLa cells were transfected with expression plasmids encoding the FLAG-tagged proteins as indicated. Mitochondria were stained using MitoTracker Deep Red FM (in *blue*). The cells were immunolabeled with anti-FLAG and anti-FASTKD2 as an MRG-specific marker. *White boxes* indicate the regions shown at higher magnification. *Scale bars* are 10 μm.

To extend our knowledge of MRG-associated proteins and by inference of MRG function, we used a microscopy-based approach as schematically presented in Fig. 1*A*. Because by definition all MRGs contain RNA, we first generated a list of potential mitochondrial RNA-binding proteins by comparing the previously published lists of human RNA-binding proteins (16, 17) with the total human mitochondrial proteome as defined by Mitocarta 2.0 (18). This generated an initial database of 207 potential mitochondrial proteins able to bind RNA (supplemental Fig. S1), and examination of this list revealed several functional classes of proteins. As our goal was to extend our understanding of the range of processes that take place within MRGs, we selected representative members from each functional class for further analysis. To this list, we added a number of other mitochondrial proteins, which although not necessarily binding directly to RNA appeared from previous studies to play a role in mitochondrial gene expression (supplemental Fig. S1). A total of 35 potential MRG candidates were selected for which the cDNAs were generated and cloned into an expression vector incorporating a C-terminal FLAG tag. Each of the resulting constructs was then used to transfect HeLa cells to investigate the submitochondrial localization of their products. Proteins were classified as MRG components based on the formation of distinct foci and co-localization with the established MRG component FASTKD2 (19, 20) (Fig. 1*B*). From this analysis, we identified 12 new MRG-associated proteins (Table 1), including proteins involved in RNA processing (FASTKD1, PTCD1, and PTCD2), RNA modification (TFB1M, TRUB2, RPUSD3, and RPUSD4), mitoribosome assembly (ERAL1 and NOA1/C4orf14), and structural components of mitoribosome (MRPL47, MRPS7, and MRPS9). Interestingly, some of the novel MRG-associated proteins (ERAL1, PTCD1, RPUSD3, and TRUB2) also emerged to physically interact with the already known MRG protein components FASTK, FASTKD2, GRSF1, and MRPP1 in co-immunoprecipitation experiments (supplemental Fig. S2 and supplemental Table S1). From these observations, we can confirm that the MRGs contain proteins participating in almost all aspects of mitochondrial gene expression.

**TABLE 1:** **List of novel MRG components** FAST, Fas-activated serine/threonine.

Symbol	Gene name	Function
*ERAL1*	GTPase Era, mitochondrial tRNAs	Mitoribosome assembly
*FASTKD1*	FAST kinase domain-containing protein 1	RNA processing
*MRPL47*	Mitochondrial ribosomal protein L47	Ribosomal subunit
*MRPS7*	Mitochondrial ribosomal protein S7	Ribosomal subunit
*MRPS9*	Mitochondrial ribosomal protein S9	Ribosomal subunit
*NOA1* (C4orf14)	Nitric oxide-associated protein 1	Mitoribosome assembly
*PTCD1*	Pentatricopeptide repeat-containing protein 1, mitochondrial	RNA processing
*PTCD2*	Pentatricopeptide repeat-containing protein 2, mitochondrial	RNA processing
*RPUSD3*	RNA pseudouridylate synthase domain-containing protein 3	RNA modification
*RPUSD4*	RNA pseudouridylate synthase domain-containing protein 4	RNA modification
*TFB1M*	Dimethyladenosine transferase 1, mitochondrial	RNA modification
*TRUB2*	Probable tRNA pseudouridine synthase 2	RNA modification

**FIGURE 2.**
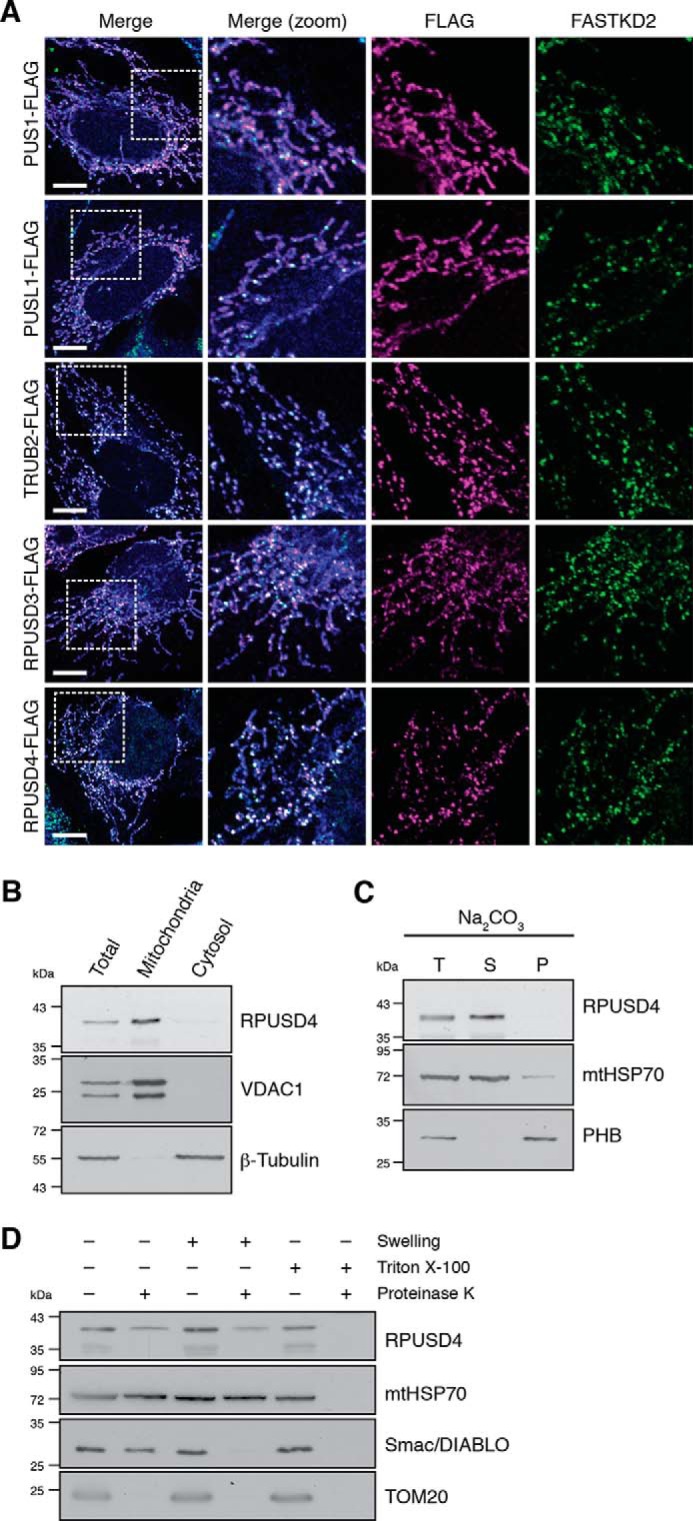
**Submitochondrial localization of the mitochondrial pseudouridine synthases.** *A*, confocal analysis of each of the five mitochondrial pseudouridine synthases. 143B cells were transfected with expression plasmids encoding the FLAG-tagged proteins indicated. Mitochondria were stained using MitoTracker Deep Red FM (in *blue*). The cells were immunolabeled with anti-FLAG and anti-FASTKD2 as an MRG-specific marker. *White boxes* indicate the regions shown at higher magnification. *Scale bars* are 10 μm. *B*, subcellular fractionation of 143B cells and analysis by Western blotting show enrichment for the endogenous RPUSD4 in the mitochondrial fraction. Total cell lysate (*T*), mitochondrial (*M*), and cytosolic (*C*) fractions were assessed for purity using β-tubulin and VDAC1 as cytosolic and mitochondrial markers, respectively. *C*, immunoblotting analysis of total isolated 143B mitochondria (*T*) or the supernatant (*S*) and pellet (*P*) fractions following alkaline sodium carbonate (*Na_2_CO_3_*) extraction. mtHSP70 and prohibitin (*PHB*) are markers for the mitochondrial matrix and mitochondrial membranes, respectively. *D*, immunoblot analysis of isolated 143B mitochondria after proteinase K (*PK*) accessibility test. Mitochondria were left untreated, swollen to rupture of the outer membrane, or lysed in Triton X-100 prior to treatment with proteinase K. mtHSP70 is a mitochondrial matrix protein, Smac/DIABLO is an intermembrane space protein, and TOM20 is a mitochondrial outer membrane protein.

From the list of the proteins specifically associated with MRGs, we selected three poorly characterized proteins, TRUB2, RPUSD3, and RPUSD4, which belong to a group of five mitochondrial pseudouridine synthases (see the Introduction). To determine whether MRG localization is a general feature of this protein family, we investigated the submitochondrial localization of each of the five enzymes (TRUB2, RPUSD3, RPUSD4, PUS1, and PUSL1) by immunofluorescence. For this, each of these enzymes was tagged with a FLAG peptide at the C terminus and expressed at a low level in 143B cells. We observed a clear enrichment of RPUSD3, RPUSD4, and TRUB2 in MRGs, whereas neither PUS1 nor PUSL1 appeared to be specifically enriched in discrete foci (Fig. 2*A*). We conclude therefore that the submitochondrial localization of the pseudouridine synthases is a specific property of each member of the family.

In the present study, we concentrated primarily on RPUSD4. To confirm the mitochondrial localization of the endogenous protein, we performed subcellular fractionation and alkaline sodium carbonate extraction. The results of these experiments demonstrated that RPUSD4 is a mitochondrial protein not associated with membranes (Fig. 2, *B* and *C*). In agreement with its MRG localization, proteinase K digestion showed that the endogenous RPUSD4 was protected from digestion in intact mitochondria and mitoplasts but not in permeabilized mitochondria (Fig. 2*D*). Taken together, these data show that RPUSD4 is a mitochondrial matrix protein associated with mitochondrial RNA granules.

To study the function of RPUSD4, our initial approach was to disrupt the gene in 143B cells using the CRISPR/Cas9 technology. However, we were unable to obtain a complete homozygous knock-out, suggesting that *RPUSD4* could be an essential gene. In agreement with this conclusion, *RPUSD4* was among the recently published list of human essential genes (21). Nevertheless, we obtained two viable clonal lines, both of which were found to carry a nonsense mutation in one allele together with a second mutation in the other allele. In one case, this was a point mutation, and in the other it was a 21-nt deletion (supplemental Fig. S3A). Both mutations are in exon 1 in the region encoding the mitochondrial targeting signal, although neither prevented mitochondrial localization of the mutant protein (supplemental Fig. S3, A and B). In agreement with the genotype, we observed a reduction of RPUSD4 protein level of about 50% (supplemental Fig. S3B).

**FIGURE 3.**
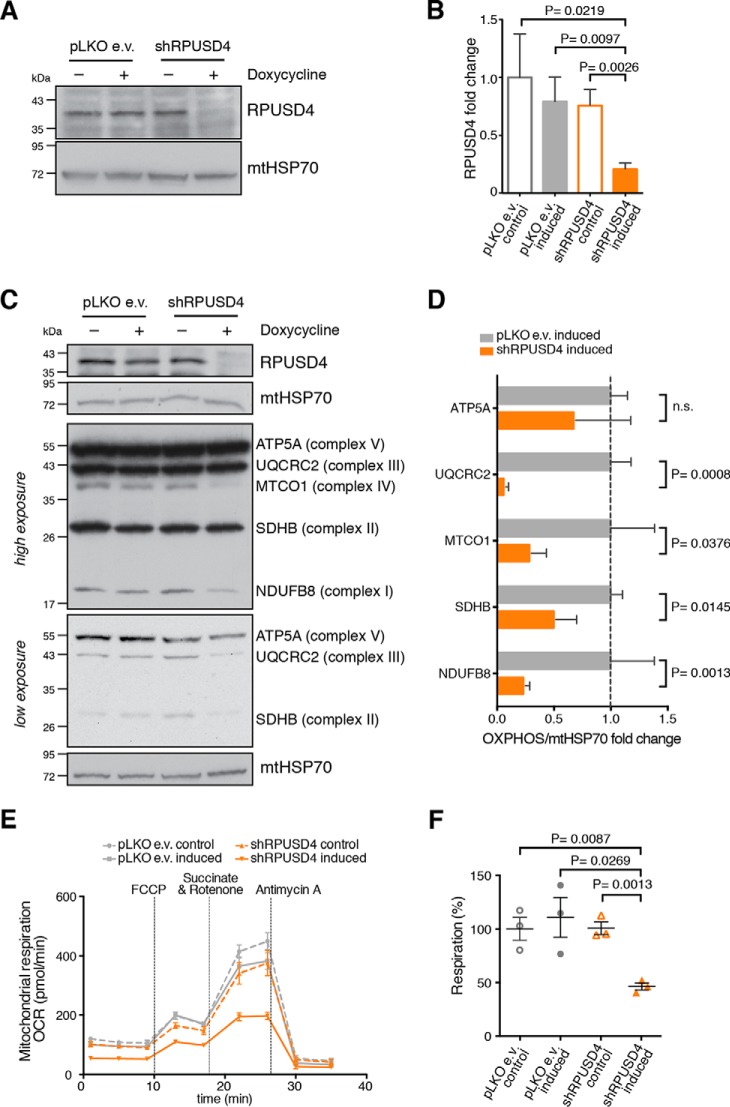
**RPUSD4 is required for mitochondrial respiration.** *A*, shRNA-mediated knockdown of RPUSD4. Immunoblotting analysis shows a decreased level of RPUSD4 after 7-day induction (100 ng/ml doxycycline) of shRNA (shRPUSD4). The empty vector (pLKO e.v.) was used as a control for doxycycline treatment, and mtHSP70 was used as a loading control. *B*, densitometric quantification of the immunoblot shown in *A*. RPUSD4 values were normalized to mtHSP70 and expressed as -fold change relative to the pLKO control. Data for the mean values are from three independent experiments; *error bars* represent S.D. *p* values were calculated using Student's *t* test. *C*, SDS-PAGE analysis shows reduced steady-state levels of respiratory chain components in RPUSD4-silenced 143B cells. Western blotting membranes were probed with an antibody mixture specific for members of each OXPHOS complex. mtHSP70 and β-tubulin were used as loading controls. Due to differences in the signals for the respiratory chain subunits, high and low exposures of the blot are presented. *D*, densitometric quantification of the data shown in *C*. The values obtained for each of the OXPHOS subunits were normalized to the values obtained for mtHSP70 and expressed as -fold change relative to the pLKO e.v. control cells treated with doxycycline. Data were obtained from three independent experiments, and means are indicated; *error bars* represent S.D. *p* values were calculated using Student's *t* test. *E*, RPUSD4 silencing impairs mitochondrial respiration. Representative profiles of oxygen consumption rates (*OCR*) in the XF Plasma Membrane Permeabilizer reagent-permeabilized cell lines are indicated. Assays were performed in the presence of pyruvate (10 mm) and malate (1 mm) as carbon sources, and the following compounds were injected at the times indicated by *vertical lines*: carbonyl cyanide-4-(trifluoromethoxy)phenylhydrazone (*FCCP*; 2 μm), succinate (10 mm) and rotenone (1 μm), and antimycin A (1 μm). All measurements were made in the presence of oligomycin (1 μm) to inhibit ATP-linked respiration. Data are represented as means ± S.D. *F*, oligomycin-sensitive respiration was normalized to antimycin A-driven respiration and expressed as percentage of respiration relative to control (pLKO e.v.). Data from three independent experiments are represented as means; *error bars* represent S.E. *p* values were obtained using Student's *t* test. *SDH*, succinate dehydrogenase.

In the light of these findings, we decided to pursue an alternative approach in which we performed inducible shRNA-mediated knockdown of *RPUSD4* in 143B cells. Upon induction of the shRNA expression, we obtained ∼80% reduction in the steady-state level of the RPUSD4 protein (Fig. 3, *A* and *B*). To investigate the effect of RPUSD4 depletion on mitochondrial function, we first analyzed the steady-state level of a set of OXPHOS subunits in *RPUSD4*-silenced cells by Western blotting and observed an overall reduction in the subunits of respiratory complexes in RPUSD4-depleted cells (Fig. 3, *C* and *D*). We then measured the oxygen consumption rate in permeabilized cells silenced for *RPUSD4* and in control cells. Consistent with the previous result, we observed a decrease in OXPHOS activity (Fig. 3, *E* and *F*). Interestingly, the clones in which RPUSD4 was partially depleted through CRISPR/Cas9 mutagenesis also showed reduced levels of respiratory complex subunits, which could be restored by overexpression of RPUSD4 (supplemental Fig. S3C), and reduced respiratory activity (supplemental Fig. S3, D and E). Together, these experiments reveal that RPUSD4 is necessary for mitochondrial respiration.

**FIGURE 4.**
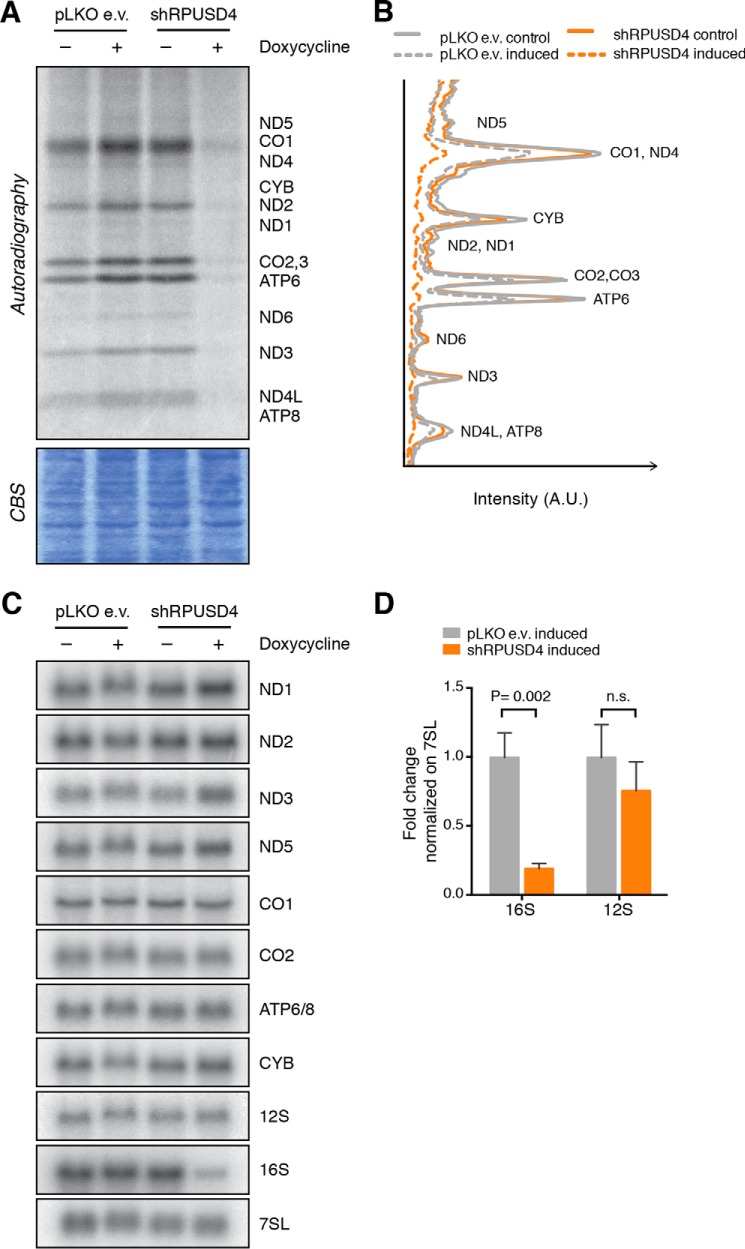
**Silencing of RPUSD4 affects mitochondrial protein translation by decreasing the 16S ribosomal RNA.** *A*, ^35^S labeling of *de novo* mitochondrial protein synthesis in RPUSD4-silenced 143B cells with a region of the Coomassie Blue-stained gel (*CBS*) shown as a loading control. *B*, phosphorimaging quantification profile of the bands shown in *A. C*, Northern blotting analysis of total RNA extracts from RPUSD4-silenced 143B cells (shRPUSD4) and control cells (pLKO e.v.) probed for the RNA sequences as indicated. Cytosolic 7SL RNA was used as a loading control. *D*, densitometric quantification of the results for the rRNAs shown in *C*. The values for 16S and 12S rRNAs were normalized to the value obtained for the 7SL RNA and expressed as the -fold change with respect to the value obtained for pLKO e.v. cells treated with doxycycline. The combined data from four independent experiments are shown as means; *error bars* represent S.D. *p* values were obtained using Student's *t* test. *CYB*, cytochrome *b*.

We next investigated whether the impairment of OXPHOS in cells depleted for RPUSD4 was due to a defect in mitochondrial gene expression. Mitochondrial protein synthesis was monitored in 143B cells following incorporation of [^35^S]methionine and [^35^S]cysteine and revealed a marked decrease in global mitochondrial translation in RPUSD4-depleted cells (Fig. 4, *A* and *B*).

To determine whether this decrease in mitochondrial protein synthesis could be due to a defect in transcription and/or mtRNA processing, we analyzed the steady-state level of a representative subset of mitochondrial transcripts by Northern blotting. No significant effect on the levels of mitochondrial protein coding mt-mRNAs was detected (Fig. 4*C*). In contrast, we observed a marked reduction in the level of 16S mt-rRNA in RPUSD4-depleted cells even though the level of 12S mt-rRNA was unaffected (Fig. 4*D*). Taken together, these results argue against the hypothesis that the reduced mitochondrial translation in RPUSD4-depleted cells is due to a global defect in the synthesis or processing of polycistronic transcripts but rather suggest a specific role of RPUSD4 in stabilizing the 16S mt-rRNA.

**FIGURE 5.**
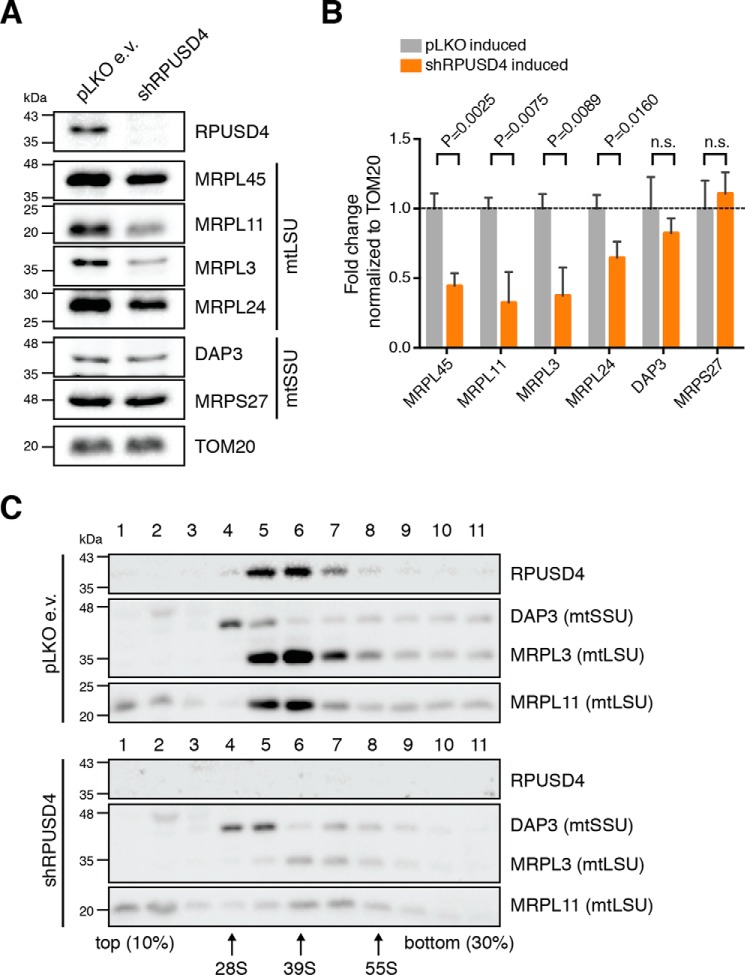
**RPUSD4 co-migrates with the mt-LSU and is essential for its biogenesis.** *A*, immunoblotting analysis of the steady-state levels of several MRPs in 143B cells expressing shRPUSD4 or pLKO e.v. following doxycycline induction. Western blotting membranes were probed with antibodies against proteins of the mt-LSU or mt-SSU as indicated. TOM20 was used as a loading control. *B*, densitometric quantification of the data shown in *A*. MRP values were normalized to TOM20 values and expressed as the -fold change with respect to the pLKO e.v. control cells treated with doxycycline. Data from three independent experiments are represented as means; *error bars* represent S.D. *p* values were obtained using Student's *t* test. *C*, sucrose gradient sedimentation to analyze the distribution of RPUSD4 and mitoribosomal proteins. Cell lysates of doxycycline-induced 143B cells expressing the pLKO e.v. (*top*) or shRPUSD4 (*bottom*) were separated through 10–30% (w/v) isokinetic sucrose gradients and fractionated. The fractions were analyzed by Western blotting using antibodies specific for endogenous RPUSD4, a component of mt-LSU (MRPL3 or MRPL11), or a component of mt-SSU (DAP3).

Because the silencing of RPUSD4 caused a substantial decrease in the level of the 16S mt-rRNA, we hypothesized that RPUSD4 could be involved in the assembly of the large 39S mt-LSU. To test this, we analyzed the steady-state levels of representative mitoribosomal proteins (MRPs). Western blotting revealed a marked reduction in the steady-state levels of 39S mt-LSU proteins MRPL45, MRPL11, MRPL3, and MRPL24, whereas no differences were observed for the 28S small subunit (mt-SSU) proteins DAP3 and MRPS27 (Fig. 5, *A* and *B*). To investigate the consequences on the mt-LSU, we performed isokinetic sucrose gradients on control and *RPUSD4*-silenced 143B cell lysates. In control cells, RPUSD4 was found to co-sediment specifically with the mt-LSU but not with the mt-SSU (Fig. 5*C*). However, when RPUSD4 was silenced, we observed a marked reduction in the levels of the mt-LSU assembled with no major changes in the sedimentation profile of the mt-SSU (Fig. 5*C*). These results, together with the decrease of the 16S mt-rRNA level, suggest that RPUSD4 may interact directly with the 39S mt-LSU, playing an essential role in its assembly and/or stability possibly through the pseudouridylation of the 16S mt-rRNA.

**FIGURE 6.**
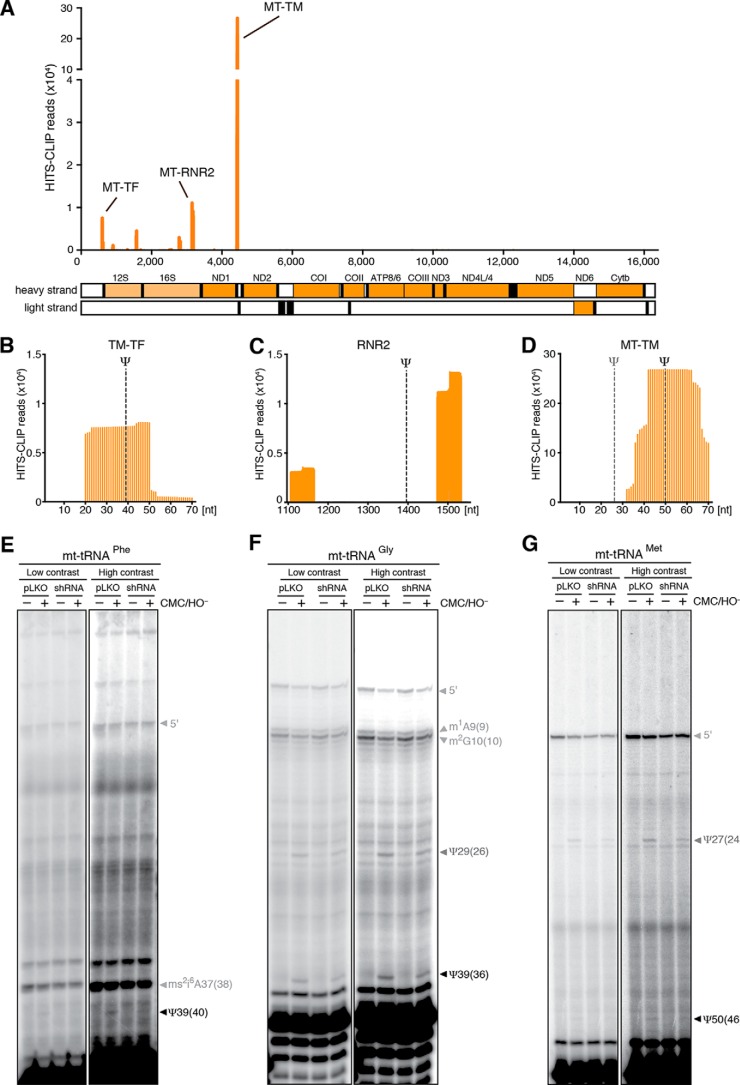
**RPUSD4 interacts with the 16S rRNA, mt-tRNA^Met^, and mt-tRNA^Phe^ and modifies the latter.** *A*, HITS-CLIP reads mapped to mtDNA represented on the *x axis* (mt-tRNA in *black*, mt-mRNAs in *dark orange*, and mt-rRNAs in *light orange*). *B–D*, detailed view of the reads mapped to mt-tRNA^Phe^ (*TM-TF*), 16S (*RNR2*), and the mt-tRNA^Met^ (*TM-MT*) regions. On the *x axis* are the nucleotide positions for each gene. *Dashed lines* indicate the known pseudouridylation sites (the putative RPUSD4 pseudouridylation sites are indicated in *black*, and the others are in *dark gray*). *E–G*, primer extension experiments on RNA treated with CMC/OH^−^ to map pseudouridines. Total RNA from pLKO e.v.- or shRPUSD4 (shRNA)-expressing cells was treated with CMC/OH^−^ and used to perform reverse transcription primer extension using specific primers for mt-tRNA^Phe^ (*E*), mt-tRNA^Gly^ (*F*), and mt-tRNA^Met^ (*G*). Positions of pseudouridine and other RNA modifications are indicated for each panel (the putative pseudouridylated sites are indicated in *black*, the other pseudouridines are in *dark gray*, and all the other modifications are indicated in *light gray*). Low and high contrast images are presented to facilitate the visualization of low intensity signals.

To provide further insight into possible RPUSD4 RNA substrates, we performed UV cross-linking and immunoprecipitation experiments followed by high throughput RNA sequencing (HITS-CLIP), on the lysate of 143B cells expressing a FLAG-tagged version of RPUSD4. A highly selective subset of mitochondrial transcripts was found to interact with RPUSD4, including most prominently mt-tRNA^Phe^, the 16S mt-rRNA, and mt-tRNA^Met^ (Fig. 6*A*). Because HITS-CLIP generates protected sequence tags of about 30 nt, we were able to map the RPUSD4 binding sites in each of these transcripts. For the mt-tRNA^Phe^ gene (*MT-TF*) and mt-tRNA^Met^ gene (*MT-TM*), this analysis revealed a clear enrichment for the regions containing the pseudouridine at universal tRNA sites 39 and 50, respectively (Fig. 6, *B* and *D*). In contrast, the majority of the reads obtained for the 16S gene (encoded by *RNR2* gene) did not map to the region corresponding to the known Ψ1397 site but to a site ∼100 nt further downstream (Fig. 6*C*). To extend the analysis of RPUSD4 activity on the two identified mt-tRNAs, we performed primer extension experiments in the presence of *N*-cyclohexyl-*N*′-(2-morpholinoethyl)carbodiimide metho-*p*-toluenesulfonate (CMC) followed by alkali treatment, which is known to selectively modify Ψ, blocking the reverse transcription (22). We could detect a significant reduction of Ψ39 in the mt-tRNA^Phe^ when RPUSD4 was depleted (Fig. 6*E*). As a control, we analyzed the mt-tRNA^Gly^, which also presents a pseudouridine at universal position 39; however, no differences were observed for this mt-tRNA in pseudouridylation of position 29 and, more interestingly, position 39 (Fig. 6*F*), consistent with the HITS-CLIP results that did not reveal any RPUSD4/mt-tRNA^Gly^ interaction. Finally, we analyzed the mt-tRNA^Met^, which was found greatly enriched in our RPUSD4 HITS-CLIP experiment. The mt-tRNA^Met^ contains two different pseudouridylation sites: Ψ27, which is known to be modified by PUS1 enzyme (10, 13), and Ψ50 for which the responsible enzyme is still unidentified (10). We could not observe any differences in the modification of Ψ27 or of Ψ50 for this mt-tRNA in *RPUSD4*-silenced cells (Fig. 6*G*), suggesting that RPUSD4 is not the enzyme responsible for the generation of the Ψ in position 50.

**FIGURE 7.**
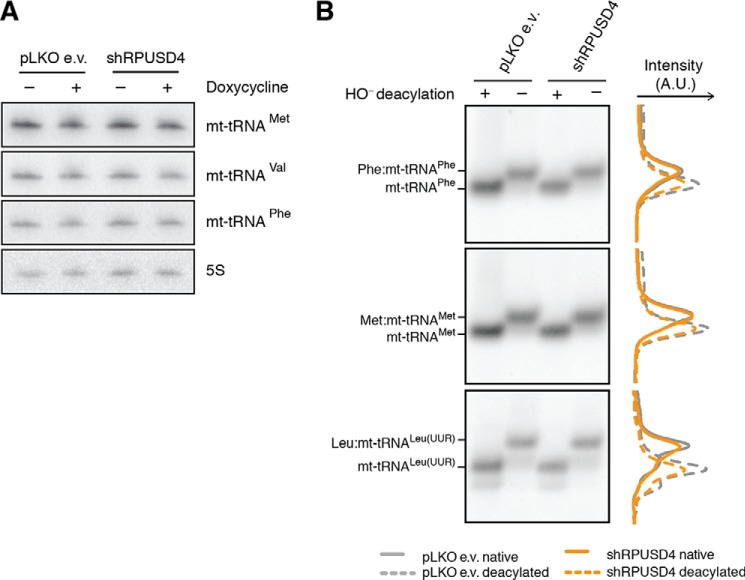
**Defective pseudouridylation of mt-tRNA^Phe^ does not affect its stability or its aminoacylation.** *A*, high resolution Northern blotting analysis of total RNA extracts from RPUSD4-silenced 143B cells (shRPUSD4) and control cells (pLKO e.v.) probed for mt-tRNA^Met^, mt-tRNA^Val^, and mt-tRNA^Phe^. Cytosolic 5S rRNA was used as a loading control. *B*, analysis of aminoacyl mt-tRNAs by high resolution Northern blotting of total RNA from pLKO e.v.- or shRPUSD4-expressing cells isolated under acid conditions. Where indicated the RNA was treated with alkali (OH^−^) to deacylate tRNAs. Membranes were probed for mt-tRNA^Phe^, mt-tRNA^Met^, and mt-tRNA^Leu(UUR)^. Densitometry profiles are presented for each mt-tRNA on the *right side* of the respective blot. *A.U.*, arbitrary units.

To investigate the role of Ψ39 in mt-tRNA^Phe^, we first analyzed the steady-state level of this mt-tRNA as well as mt-tRNA^Met^ and mt-tRNA^Val^ in *RPUSD4* knockdown cells. However, we could not observe any substantial changes (Fig. 7*A*). Next, we assayed the impact of the reduced levels of Ψ39 on aminoacylation of mt-tRNA^Phe^ by high resolution Northern blotting of RNA isolated from RPUSD4-depleted cells. The use of low pH throughout the procedure allowed for distinction between the aminoacyl-tRNA and the uncharged tRNA. We did not detect any substantial changes in the ratio between the aminoacylated and deacylated forms of mt-tRNA^Phe^ (Fig. 7*B*). Also, no changes in aminoacylation were detected in control mt-tRNA^Met^ and mt-tRNA^Leu(UUR)^ (Fig. 7*B*). Taken together, we conclude that RPUSD4 physically interacts with mt-tRNA^Phe^, 16S mt-rRNA, and mt-tRNA^Met^. We also demonstrate that RPUSD4 is responsible for the formation of Ψ39 in mt-tRNA^Phe^ and provide evidence that this modification is not crucial for the regulation of steady-state levels or aminoacylation of mt-tRNA^Phe^.

## Discussion

To identify novel protein components of RNA granules, we used a microscopy-based approach to screen a group of mitochondrial proteins potentially involved in different aspects of mitochondrial gene expression. Twelve proteins of different functional classes have been specifically localized to these structures and represent newly described components of MRGs. The functional diversity of these MRG components supports the earlier conclusion that multiple steps in mitochondrial gene expression take place within this compact submitochondrial compartment (15). The newly identified proteins are implicated in mtRNA processing (FASTKD1, PTCD1, and PTCD2), mtRNA modification (TFB1M, TRUB2, RPUSD3, and RPUSD4), mitoribosome assembly (ERAL1 and NOA1/C4orf14), and mitoribosome structure (MRPL47, MRPS7, and MRPS9). This expands the previously established list of MRG-associated proteins that includes GRSF1 (23, 24), FASTK (20), FASTKD2 (19, 20), FASTKD5 (19), MRPP1 (23, 25), DDX28 (26), MRM1–3 (27, 28), and MTPAP (29). Thus, we provide further evidence that MRGs are intimately involved in all stages of mitochondrial gene expression from the accumulation of newly synthesized RNA (14, 23) to the assembly of the mitoribosomes (19, 26, 30). Interestingly, our microscopy-based analysis has also revealed that numerous other proteins are partially enriched in the RNA granules while also being located within the mitochondrial matrix, underlining the probable dynamic nature of MRG protein composition. These conclusions are based on overexpression studies that may lead to a wider distribution pattern for certain proteins, although care was taken to image only cells with low levels of recombinant protein expression. The distribution of proteins that appear to partition between MRGs and the matrix will be confirmed in the future by analysis of the endogenous proteins using specific antibodies.

Among the newly identified MRG components, we focused on three hitherto poorly characterized putative mitochondrial pseudouridine synthases, RPUSD3, RPUSD4, and TRUB2. Consistent with our results showing specific localization of these three proteins to MRGs, Arroyo *et al*. (31) showed recently that these same three proteins together with FASTKD2, Neugrin (NGRN), and WBSCR16 constitute a “functional module” that is able to regulate the level of 16S mt-rRNA and, more generally, the capacity for mitochondrial translation. Interestingly, two other members of the mitochondrial pseudouridine synthase family, PUS1 and PUSL1, do not appear to localize specifically to MRGs. However, as discussed above, definitive conclusions in this case must await analysis of the endogenous proteins.

In this study, we focused on RPUSD4, and we provide the first detailed characterization of this protein. Using a CRISPR/Cas9 gene disruption approach, we found that 143B cells carrying a homozygous deletion of *RPUSD4* are not viable, suggesting an essential role of this protein in cell survival. To investigate the function of RPUSD4 more in detail, we used an inducible shRNA approach, which reduced the protein level to ∼20% of control. Under these conditions, we observed a significant decrease in the levels of certain subunits of the OXPHOS system and a substantial reduction in the respiratory capacity of cells. A similar phenotype was observed in the CRISPR/Cas9-generated heterozygous mutants in which *RPUSD4* expression was reduced to approximately 50%. This phenotype could be rescued by re-expression of RPUSD4 to physiological levels in the heterozygous cells, thus demonstrating that the observed reduction in mitochondrial respiration is specific to the heterozygosity. These data are in full agreement with the recent CRISPR/Cas9 screen performed by Arroyo *et al.* (31), indicating that *RPUSD4* is among the genes essential for OXPHOS.

Here we show that the respiratory defect in RPUSD4-depleted cells is a consequence of impaired mitochondrial translation and not the result of a general defect in expression of mt-RNAs. We found that the only mtRNA to be affected by knockdown of *RPUSD4* is the 16S mt-rRNA with no significant effect exerted on the levels of the 12S mt-rRNA and mt-tRNA^Val^, which are both present on the same polycistronic mtRNA precursor, or on any of the other mt-tRNAs examined. From these results, we hypothesized that the observed phenotype was most likely due to increased degradation of the 16S mt-rRNA rather than to a global defect in synthesis or processing of the polycistronic mtRNA. Furthermore, our analysis of mitoribosome biogenesis indicated specific impairment of the mt-LSU, consistent with our data that RPUSD4 was found to co-migrate with the mt-LSU but not with the mt-SSU. Finally, our HITS-CLIP analysis demonstrated a direct interaction of RPUSD4 with the 16S mt-rRNA. Taken together, these results suggested that RPUSD4 is responsible for pseudouridylation of the 16S mt-rRNA, and this constitutes an essential step in the assembly of the mitoribosome. In the absence of RPUSD4, the 16S mt-rRNA would not be pseudouridylated, and consequently correct assembly of the mt-LSU would be impaired, leading to the degradation of its components. Although the 16S mt-rRNA was previously found to be pseudouridylated at position 1397 (11, 12), our HITS-CLIP analysis revealed that RPUSD4 binds at a site ∼100 nt downstream of this site. Interestingly, the three-dimensional model of 16S mt-rRNA proposed by Greber *et al.* (32) shows that the region we found directly associated with RPUSD4 is in close proximity to the domain where the pseudouridylation site resides. In agreement with our results, Antonicka *et al.* (33) very recently showed that RPUSD4 plays a direct role in the modification of the 16S mt-rRNA at position 1397.

In addition to the 16S mt-rRNA, our HITS-CLIP data identified a further two binding sites. These revealed mt-tRNA^Phe^ and mt-tRNA^Met^ as specific interactors for RPUSD4. To confirm these data and to investigate a direct role of RPUSD4 in modifying these two mt-tRNAs, we performed primer extension analyses following treatment with CMC and alkali, which modifies pseudouridines and prevents readthrough of the reverse transcriptase. We were able to demonstrate that RPUSD4 is responsible for the formation of Ψ39 on mt-tRNA^Phe^. This appears to be specific for mt-tRNA^Phe^ because no changes in modification of Ψ39 on mt-tRNA^Gly^ were observed. Curiously, despite the greatly larger number of reads mapped in our HITS-CLIP for mt-tRNA^Met^ at precisely the Ψ50 site, depletion of RPUSD4 did not result in loss of pseudouridylation at this site. This raises the possibility that RPUSD4 may interact physically with mt-tRNA^Met^ without being the enzyme directly involved in its modification. Thus, we suggest that selectivity of RPUSD4 for its target mt-tRNA is determined not only by the position of the residue within the mt-tRNA structure but also by sequence differences around the region of pseudouridylation. Despite the reduced levels of Ψ39 in the mt-tRNA^Phe^, we did not observe any differences in the steady-state level of this mt-tRNA, suggesting that Ψ39 does not play a role in the stability of this mt-tRNA. Moreover, we did not observe any substantial differences in its aminoacylation in RPUSD4-depleted cells. Thus, the significance of the Ψ39 modification on mt-tRNA^Phe^ still requires further investigation.

The mammalian pseudouridine synthases are generally classified into five families, each related to one of the five *Escherichia coli* pseudouridine synthases (TruA, TruB, TruD, RsuA, and RluA), which are responsible for the modification of different RNA substrates (6, 34). In human mitochondria, the pseudouridine synthases PUS1 and PUSL1 belong to the TruA family, and TRUB2 belongs to the TruB family, whereas RPUSD3 and RPUSD4 are members of the RluA family. Interestingly, the bacterial protein RluA is the only pseudouridine synthase that displays a dual specificity, being able to modify both the large 23S rRNA and several tRNAs, including tRNA^Phe^ in position 32 (35). In light of our data on RPUSD4, we suggest that pseudouridylation in mammalian mitochondria has been highly conserved throughout evolution from bacteria to human.

## Experimental Procedures

### 

#### 

##### Cell Culture, Transfection, and Chemicals

All cell culture reagents and chemicals were purchased from Sigma unless stated otherwise. Cells were cultured in Dulbecco's modified Eagle's medium (DMEM) supplemented with 10% heat-inactivated fetal bovine serum (FBS), 100 units/ml penicillin, 100 μg/ml streptomycin, and 2 mm l-glutamine in 5% CO_2_ at 37 °C. CRISPR/Cas9 cells were grown in the same medium supplemented with 110 μg/ml sodium pyruvate and 50 μg/ml uridine. Transfection of cells was performed using Lipofectamine LTX (Invitrogen) according to the manufacturer's instructions, and cells were analyzed 16–24 h after transfection.

##### Generation of CRISPR/Cas9 Cells

To generate RPUSD4 knock-out cells using the CRISPR/Cas9 technology, we cloned the following guide RNA into the pSpCas9(BB)-2A-GFP (pX458) vector (Addgene): sense, CAC CTT GGC CGT TTC CCC GGA TCC; antisense, AAA CGG ATC CGG GGA AAC GGC CAA. 143B cells were transfected using Lipofectamine LTX (Invitrogen). 48 h after transfection, GFP^+^ cells were selected by fluorescence-activated cell sorting (FACS) using the Beckman Coulter MoFlo Astrios, and single cells were transferred to individual wells of a 96-well plate to obtain single cell clones. Screening of clones was performed by Sanger sequencing of the RPUSD4 gene and by Western blotting analysis.

##### Cloning, Viral Production, and RNA Interference

Cloning the cDNAs for potential MRG proteins was performed by RT-PCR using a human RNA library derived from different tissues as template. Briefly, individual cDNAs for each gene were generated by reverse transcription using a pool of ∼10 gene-specific primers located immediately downstream of the coding sequence, and aliquots of the cDNA pool were PCR-amplified using primers designed to generate the full-length cDNA. Unique restriction sites were added to the 5′ extremities of each PCR primer, and the cDNAs were cloned into the pCi mammalian expression vector (Promega) in-frame with a C-terminal FLAG tag. For lentiviral expression, the RPUSD4 cDNA (transcript variant 1) was subcloned together with the C-terminal FLAG tag into the constitutive lentiviral expression vector pWPT (Addgene). Stable cell lines were generated using a lentivirus-based procedure. Briefly, the cDNA was cloned into pWPT and co-transfected with the lentiviral packaging plasmids pMD2.G and psPAX2 (Addgene) into HEK293T cells by calcium phosphate co-precipitation. Medium containing virus was collected 48 h after transfection and filtered using membranes with a pore size of 0.45 μm. The viral supernatant was added to 70% confluent recipient cells, and transduction efficiency was estimated by immunofluorescence and Western blotting.

For RNA interference, the following shRNA sequence against RPUSD4 (shRPUSD4) was cloned into the pLKO-Tet-On vector (Addgene): sense, 5′-CCG GGC TTC GAG TTC ACT TGT CCT TCT CGA GAA GGA CAA GTG AAC TCG AAG CTT TTT G; antisense, 5′-AAT TCA AAA AGC TTC GAG TTC ACT TGT CCT TCT CGA GAA GGA CAA GTG AAC TCG AAG C. The pLKO-Tet-On empty vector was always included as a negative control. Production of lentivirus was performed as described above. After 48 h of infection, cells were selected with 5 μg/ml puromycin for a further 48 h. Induction of shRNA expression was achieved following addition of 100 ng/ml doxycycline for 7 days.

##### Protein Co-immunoprecipitation and Mass Spectrometry

A mitochondrion-rich fraction was isolated from 143B cells stably expressing either GFP (as a control), the mitochondrial version of FASTK-FLAG (20), FASTKD2-FLAG, GRSF1-FLAG, or MRPP1-FLAG, and cross-link-free RNase-free FLAG immunoprecipitation was performed as described previously (31). Proteins were eluted with a 3xFLAG peptide (Sigma), precipitated with trichloroacetic acid (TCA), and analyzed by nano-LC-MS-MS at the proteomics core facility of the University of Geneva. True hits were scored based on the identification of at least three peptides per protein, an enrichment of minimum 2-fold over the GFP control (scaffold) and based on their presence in MitoCarta2.0 (18).

##### Immunofluorescence and Microscopy

Immunofluorescence analyses were performed on cells fixed in 4% paraformaldehyde for 15 min. Cell permeabilization and blocking were done together by incubating the fixed cells in PBS containing 0.3% Triton X-100 and 1% preimmune goat serum for 1 h. The same buffer was used to incubate cells with the specified primary antibody (see antibody section below). After 2-h incubation, the cells were washed in PBS and incubated with the appropriate secondary antibody conjugated with Alexa Fluor 488 or Alexa Fluor 594 (Life Technologies). Mitochondrial networks were stained before fixing the cells using MitoTracker Deep Red FM (Thermo Fisher Scientific) according to the manufacturer's instructions. Imaging was performed using a Zeiss LSM700 confocal microscope equipped with a 63× oil objective. All images were imported in ImageJ and uniformly adjusted for brightness and contrast.

##### Mitochondrion-rich Fraction Isolation, Proteinase K Accessibility Test, and Alkali Treatment

Preparation of cytosolic, nuclear, and mitochondrion-rich fractions; alkali treatment; and proteinase K accessibility assays were performed as described previously (23).

##### Western Blotting

Unless otherwise specified, cell lysates were prepared by lysing cells in radioimmune precipitation assay lysis buffer (150 mm NaCl, 1% Triton X-100, 0.5% sodium deoxycholate, 0.1% SDS, and 50 mm Tris, pH 8.0) for 30 min on ice followed by removal of insoluble material by centrifugation at 16,000 × *g* for 10 min at 4 °C. Protein content was determined using the Bradford protein assay (Bio-Rad), and equal amounts of protein were analyzed by SDS-PAGE. For immunoblotting, proteins were transferred electrophoretically to nitrocellulose or PVDF membranes (GE Healthcare) and exposed to the specified primary antibodies (see antibody section below). The blots were further incubated with anti-goat, anti-rabbit, or anti-mouse HRP-conjugated secondary antibodies (Dako) and visualized using ECL. Where required, images of Western blotting were treated for contrast enhancement and quantified using ImageJ.

##### Determination of Respiratory Chain Activity

Measurement of oxygen consumption was performed using a Seahorse XF^e^24 Flux Analyzer (Seahorse Biosciences). 35,000 cells were seeded into XF24 cell culture microplates and grown overnight in DMEM containing 10% FBS, 2 mm l-glutamine, 25 mm glucose, and penicillin/streptomycin. Experiments were carried out at 37 °C in Mitochondrial Assay Solution (containing 70 mm sucrose, 220 mm mannitol, 10 mm KH_2_PO_4_, 5 mm MgCl_2_, 2 mm Hepes, 1 mm EGTA, and 0.2% fatty acid-free BSA, pH 7.2). Cells were permeabilized with 1 nm XF Plasma Membrane Permeabilizer reagent (Seahorse Bioscience) and provided with 5 mm pyruvate, 0.5 mm malate, 2 mm dichloroacetate, and 1 μm oligomycin for 1 h before the assay. Basal oxygen consumption was measured before further treatment. At the times indicated, the following compounds were injected: carbonyl cyanide-4-(trifluoromethoxy)phenylhydrazone (final concentration, 2 μm), succinate/rotenone (10 mm/1 μm), and antimycin A (1 μm). Each measurement loop consisted of 30 s mixing, 1 min waiting, and 2 min measuring oxygen consumption.

##### RNA Extraction and Northern Blotting

RNA extraction and Northern blotting analyses were performed as described previously (23). Briefly, total RNA was extracted using TRIzol (Thermo Fisher Scientific). 5–10 μg of RNA were separated on a denaturing formaldehyde 1% agarose gel for mRNAs and rRNAs or 10% polyacrylamide gel containing 7 m urea for tRNAs, transferred to a nylon membrane (GE Healthcare), and UV-crosslinked to the membrane. Membranes were hybridized with T7-transcribed [α-^32^P]UTP- radiolabeled riboprobes. Hybridization was performed at 60 °C in 50% formamide, 7% SDS, 0.2 m NaCl, 80 mm sodium phosphate, pH 7.4, and 100 μg/ml salmon sperm DNA. Imaging and quantification were performed with a phosphorimaging system (Bio-Rad) or Typhoon imaging system (GE Healthcare). A detailed list of primers used to transcribe the riboprobes is provided in supplemental Table S2.

##### Pulse Labeling of Mitochondrial Translation

Pulse labeling experiments to evaluate mitochondrial translation were performed as described previously (23). Briefly, 143B cells were incubated for 20 min in methionine- and cysteine-free DMEM (Sigma) supplemented with 10% dialyzed serum and 2 mm l-glutamine. Emetine dihydrochloride (100 μg/ml) was added for 5 min to inhibit cytosolic translation followed by addition of 100 μCi/ml S^35^-labeled methionine and cysteine mixture (PerkinElmer Life Sciences). Labeling was performed for 1 h, and then cells were lysed. The protein content of each sample was measured using the Bradford assay (Bio-Rad), and 50 μg of each protein sample were resolved by 15–20% SDS-PAGE. Gels were stained with Coomassie Brilliant Blue to confirm equal loading, then dried, and exposed. Imaging and quantification were performed with a phosphorimaging system (Bio-Rad).

##### Isokinetic Sucrose Gradients for Mitoribosome Fractionation

Isokinetic sucrose gradients were performed as described previously (36). Briefly, cell lysates (0.5–0.7 mg in lysis buffer) were loaded on to a 10–30% (v/v) linear sucrose gradient (1 ml) in 50 mm Tris-HCl, pH 7.2, 10 mm magnesium acetate, 40 mm NH_4_Cl, 0.1 m KCl, 1 mm PMSF, and 50 μg/ml chloramphenicol. Gradients were centrifuged at 39,000 × *g* for 135 min at 4 °C using a Beckman Optima TLX bench ultracentrifuge equipped with a TLS55 rotor. Fractions (100 μl) were collected, and proteins were precipitated with TCA and analyzed by Western blotting.

##### UV Cross-linking and Immunoprecipitation

HITS-CLIP was performed as described previously (36). Briefly, 143B cells expressing RPUSD4-FLAG were grown to ∼80% confluence in 4 × 15-cm plates, washed in PBS, and UV-irradiated at 400 mJ/cm^2^ in a Stratalinker (Stratagene). To ensure that only short protected RNA species remained, cells were lysed, bound ribonucleoproteins were treated with RNase T1, and RPUSD4-FLAG was immunoprecipitated using FLAG M2 magnetic beads (Sigma). Bound RNA was dephosphorylated and ligated to the 3′ linker. To visualize the RNA-protein complex, the 5′-ends were incubated with [γ-^32^P]ATP (3000 Ci/mol; PerkinElmer Life Sciences) and T4 polynucleotide kinase (New England Biolabs), separated by SDS-PAGE (10% Novex precast gels), transferred to nitrocellulose (BA-85 Whatman), and subjected to autoradiography. For RNA isolation, ribonucleoprotein complexes were cut from the nitrocellulose, protein was degraded with proteinase K, and the RNA was precipitated following phenol/chloroform extraction. Ligation of the 5′ linker, reverse transcription, and PCR amplification were performed as described previously (36). The library of PCR products was prepared according to the manufacturer's instructions (Illumina) and subjected to high throughput sequencing using the Illumina HiSeq platform. Library preparation and sequencing were performed at Fasteris (Plan-les-Ouates, Geneva). Quality trimming and 3′-end and 5′-end adaptor clipping of sequenced reads were performed using fastx_trimmer. Reads were aligned against the human reference genome (hg38) using Bowtie2/Tophat2 and then visualized and quantified with Integrative Genomics Viewer (IGV) (Broad Institute).

##### CMC/HO^−^ Modification of RNA

Total RNA from pLKO empty vector (e.v.) and RPUSD4 shRNA 143B cells was extracted using TRIzol (Invitrogen) according to the manufacturer's instructions. After dissolution, total RNA was subjected to CMC/alkaline (HO^−^) treatment according to a modified version of Bakin and Ofengand (37). Briefly, RNA was incubated with or without 167 μm CMC for 15 min at 37 °C, precipitated, and then incubated with 50 mm Na_2_CO_3_, pH 10.4, for 2 h at 37 °C. After a final precipitation step, RNA was solubilized in water.

##### Reverse Transcription Primer Extension

Reverse transcription primer extension was performed as described previously (38). Briefly, DNA oligonucleotides were 5′-radiolabeled using [γ-^32^P]ATP and T4 polynucleotide kinase; annealed to 1 μg of CMC/HO^−^-modified RNA in a 10-μl reaction containing 10 mm Tris-HCl, pH 8.0, and 1 mm EDTA at 80 °C for 2 min; and cooled slowly in a PCR block. Subsequent primer extension reactions were performed using the Omniscript RT kit (Qiagen) according to the manufacturer's instructions. After adding 3× loading dye, reaction products were separated in a 12.5% polyacrylamide gel containing 7 m urea. Dried gels were exposed to storage phosphor screens (GE Healthcare) and scanned using a Typhoon imaging system.

##### Analysis of tRNA Aminoacylation Status

mt-tRNA aminoacylation was analyzed essentially as described previously (39). Briefly, total RNA was extracted using TRIzol (Invitrogen) according to the manufacturer's instructions with the final pellet resuspended in 10 mm NaOAc, pH 5, to preserve aminoacylation. RNA to be deacylated was resuspended in 200 mm Tris-HCl, pH 9.5, and incubated at 75 °C for 5 min; then precipitated; and resuspended in 10 mm NaOAc. Deacylated and acylation-preserved RNA samples were loaded (2× loading dye: 8 m urea, 100 mm NaAcO pH 5, 0.05% bromphenol blue, and 0.05% xylene cyanol) and separated on a gel containing 6.5% polyacrylamide, 8 m urea, and 100 mm NaAcO, pH 5, at 4 °C and then transferred and UV-fixed to positively charged nylon membranes (Hybond-N+, GE Healthcare). Membranes were hybridized with ^32^P-labeled riboprobes and washed with 1× SSC solution. Afterward, the membranes were exposed to a storage phosphor screen and imaged as described above.

##### Antibodies

The following antibodies were used in this work: FLAG M2 (Sigma, F1804), FASTKD2 (ProteinTech Group, 17464-1-AP), MRPP1 (Sigma, HPA036671), GRSF1 (Sigma, AV40382), RPUSD4 (Abcam, ab122571), VDAC1 (Santa Cruz Biotechnology, sc-8829), β-tubulin (Sigma, T4026), mtHSP70 (Thermo Fisher Scientific, MA3-028), prohibitin (Thermo Fisher Scientific, MA5-12858), Smac/DIABLO (Enzo Life Sciences, ADI-905-244-100), TOM20 (Santa Cruz Biotechnology, sc-11415), total OXPHOS human WB antibody mixture (Abcam, ab110411), MRPL45 (ProteinTech Group, 15682-1-AP), MRPL11 (Cell Signaling Technology, D68F2), MRPL3 (Abcam, ab39268), MRPL24 (ProteinTech Group, 16224-1-AP), DAP3 (Abcam, ab11928), and MRPS27 (ProteinTech Group, 17280-1-AP).

##### Statistical Analyses

All data presented are the results of at least three independent experiments and are expressed as means ± S.D. or S.E. of absolute values or percentages of control. Values were analyzed for statistical significance by Student's *t* test, and exact *p* values are reported in each graph.

## Author Contributions

Experiments were designed by S. Z., P. R.-G., K. M., C. A. P., R. N. L., Z. M. C.-L., M. M., and J.-C. M. Experiments were carried out by S. Z., P. R.-G., A. R., K. M., S. P., C. A. P., and A. A. J. Bioinformatics analyses were performed by N. H. The manuscript was written and reviewed by S. Z., P. R.-G., K. M., A. A. J., R. N. L., Z. M. C.-L., M. M., and J.-C. M. following inputs and suggestions from all the authors.

## Supplementary Material

Supplemental Data

## Footnotes

3 The abbreviations used are:

OXPHOS: oxidative phosphorylation
MRG: mitochondrial RNA granule
mt: mitochondrial
LSU: large subunit
FASTK: Fas-activated serine/threonine kinase
CRISPR: clustered regularly interspaced short palindromic repeats
nt: nucleotide(s)
MRP: mitoribosomal protein
SSU: small subunit
HITS-CLIP: cross-linking and immunoprecipitation experiments followed by high throughput RNA sequencing
CMC: *N*-cyclohexyl-*N*′-(2-morpholinoethyl)carbodiimide metho-*p*-toluenesulfonate
e.v.: empty vector
NaOAc: sodium acetate
shRPUSD4: shRNA sequence against RPUSD4.

## References

[1] Powell C. A., Nicholls T. J., and Minczuk M. (2015) Nuclear-encoded factors involved in post-transcriptional processing and modification of mitochondrial tRNAs in human disease. Front. Genet. 6, 79 25806043 10.3389/fgene.2015.00079 PMC4354410

[2] Van Haute L., Pearce S. F., Powell C. A., D'Souza A. R., Nicholls T. J., and Minczuk M. (2015) Mitochondrial transcript maturation and its disorders. J. Inherit. Metab. Dis. 38, 655–680 26016801 10.1007/s10545-015-9859-z PMC4493943

[3] Lightowlers R. N., Taylor R. W., and Turnbull D. M. (2015) Mutations causing mitochondrial disease: what is new and what challenges remain? Science 349, 1494–1499 26404827 10.1126/science.aac7516

[4] Hällberg B. M., and Larsson N. G. (2014) Making proteins in the powerhouse. Cell Metab. 20, 226–240 25088301 10.1016/j.cmet.2014.07.001

[5] Ojala D., Montoya J., and Attardi G. (1981) tRNA punctuation model of RNA processing in human mitochondria. Nature 290, 470–474 7219536 10.1038/290470a0

[6] Spenkuch F., Motorin Y., and Helm M. (2014) Pseudouridine: still mysterious, but never a fake (uridine)! RNA Biol. 11, 1540–1554 25616362 10.4161/15476286.2014.992278 PMC4615568

[7] Ge J., and Yu Y. T. (2013) RNA pseudouridylation: new insights into an old modification. Trends Biochem. Sci. 38, 210–218 23391857 10.1016/j.tibs.2013.01.002 PMC3608706

[8] Fernandez-Vizarra E., Berardinelli A., Valente L., Tiranti V., and Zeviani M. (2007) Nonsense mutation in pseudouridylate synthase 1 (PUS1) in two brothers affected by myopathy, lactic acidosis and sideroblastic anaemia (MLASA). J. Med. Genet. 44, 173–180 17056637 10.1136/jmg.2006.045252 PMC2598032

[9] Rhee H. W., Zou P., Udeshi N. D., Martell J. D., Mootha V. K., Carr S. A., and Ting A. Y. (2013) Proteomic mapping of mitochondria in living cells via spatially restricted enzymatic tagging. Science 339, 1328–1331 23371551 10.1126/science.1230593 PMC3916822

[10] Suzuki T., and Suzuki T. (2014) A complete landscape of post-transcriptional modifications in mammalian mitochondrial tRNAs. Nucleic Acids Res. 42, 7346–7357 24831542 10.1093/nar/gku390 PMC4066797

[11] Schwartz S., Bernstein D. A., Mumbach M. R., Jovanovic M., Herbst R. H., León-Ricardo B. X., Engreitz J. M., Guttman M., Satija R., Lander E. S., Fink G., and Regev A. (2014) Transcriptome-wide mapping reveals widespread dynamic-regulated pseudouridylation of ncRNA and mRNA. Cell 159, 148–162 25219674 10.1016/j.cell.2014.08.028 PMC4180118

[12] Ofengand J., and Bakin A. (1997) Mapping to nucleotide resolution of pseudouridine residues in large subunit ribosomal RNAs from representative eukaryotes, prokaryotes, archaebacteria, mitochondria and chloroplasts. J. Mol. Biol. 266, 246–268 9047361 10.1006/jmbi.1996.0737

[13] Patton J. R., Bykhovskaya Y., Mengesha E., Bertolotto C., and Fischel-Ghodsian N. (2005) Mitochondrial myopathy and sideroblastic anemia (MLASA): missense mutation in the pseudouridine synthase 1 (PUS1) gene is associated with the loss of tRNA pseudouridylation. J. Biol. Chem. 280, 19823–19828 15772074 10.1074/jbc.M500216200

[14] Iborra F. J., Kimura H., and Cook P. R. (2004) The functional organization of mitochondrial genomes in human cells. BMC Biol. 2, 9 15157274 10.1186/1741-7007-2-9 PMC425603

[15] Jourdain A. A., Boehm E., Maundrell K., and Martinou J. C. (2016) Mitochondrial RNA granules: compartmentalizing mitochondrial gene expression. J. Cell Biol. 212, 611–614 26953349 10.1083/jcb.201507125 PMC4792075

[16] Baltz A. G., Munschauer M., Schwanhäusser B., Vasile A., Murakawa Y., Schueler M., Youngs N., Penfold-Brown D., Drew K., Milek M., Wyler E., Bonneau R., Selbach M., Dieterich C., and Landthaler M. (2012) The mRNA-bound proteome and its global occupancy profile on protein-coding transcripts. Mol. Cell 46, 674–690 22681889 10.1016/j.molcel.2012.05.021

[17] Castello A., Fischer B., Eichelbaum K., Horos R., Beckmann B. M., Strein C., Davey N. E., Humphreys D. T., Preiss T., Steinmetz L. M., Krijgsveld J., and Hentze M. W. (2012) Insights into RNA biology from an atlas of mammalian mRNA-binding proteins. Cell 149, 1393–1406 22658674 10.1016/j.cell.2012.04.031

[18] Calvo S. E., Clauser K. R., and Mootha V. K. (2016) MitoCarta2.0: an updated inventory of mammalian mitochondrial proteins. Nucleic Acids Res. 44, D1251–D1257 26450961 10.1093/nar/gkv1003 PMC4702768

[19] Antonicka H., and Shoubridge E. A. (2015) Mitochondrial RNA granules are centers for posttranscriptional RNA processing and ribosome biogenesis. Cell Rep. 10, p920–p932 10.1016/j.celrep.2015.01.030 25683715

[20] Jourdain A. A., Koppen M., Rodley C. D., Maundrell K., Gueguen N., Reynier P., Guaras A. M., Enriquez J. A., Anderson P., Simarro M., and Martinou J. C. (2015) A mitochondria-specific isoform of FASTK is present in mitochondrial RNA granules and regulates gene expression and function. Cell Rep. 10, 1110–1121 25704814 10.1016/j.celrep.2015.01.063

[21] Wang T., Wei J. J., Sabatini D. M., and Lander E. S. (2014) Genetic screens in human cells using the CRISPR-Cas9 system. Science 343, 80–84 24336569 10.1126/science.1246981 PMC3972032

[22] Ofengand J., Del Campo M., and Kaya Y. (2001) Mapping pseudouridines in RNA molecules. Methods 25, 365–373 11860291 10.1006/meth.2001.1249

[23] Jourdain A. A., Koppen M., Wydro M., Rodley C. D., Lightowlers R. N., Chrzanowska-Lightowlers Z. M., and Martinou J. C. (2013) GRSF1 regulates RNA processing in mitochondrial RNA granules. Cell Metab. 17, 399–410 23473034 10.1016/j.cmet.2013.02.005 PMC3593211

[24] Antonicka H., Sasarman F., Nishimura T., Paupe V., and Shoubridge E. A. (2013) The mitochondrial RNA-binding protein GRSF1 localizes to RNA granules and is required for posttranscriptional mitochondrial gene expression. Cell Metab. 17, 386–398 23473033 10.1016/j.cmet.2013.02.006

[25] Bogenhagen D. F., Martin D. W., and Koller A. (2014) Initial steps in RNA processing and ribosome assembly occur at mitochondrial DNA nucleoids. Cell Metab. 19, 618–629 24703694 10.1016/j.cmet.2014.03.013

[26] Tu Y. T., and Barrientos A. (2015) The human mitochondrial DEAD-box protein DDX28 resides in RNA granules and functions in mitoribosome assembly. Cell Rep. 10, p854–p864 10.1016/j.celrep.2015.01.033 PMC4534351 25683708

[27] Lee K. W., Okot-Kotber C., LaComb J. F., and Bogenhagen D. F. (2013) Mitochondrial ribosomal RNA (rRNA) methyltransferase family members are positioned to modify nascent rRNA in foci near the mitochondrial DNA nucleoid. J. Biol. Chem. 288, 31386–31399 24036117 10.1074/jbc.M113.515692 PMC3829452

[28] Rorbach J., Boesch P., Gammage P. A., Nicholls T. J., Pearce S. F., Patel D., Hauser A., Perocchi F., and Minczuk M. (2014) MRM2 and MRM3 are involved in biogenesis of the large subunit of the mitochondrial ribosome. Mol. Biol. Cell 25, 2542–2555 25009282 10.1091/mbc.E14-01-0014 PMC4148245

[29] Wilson W. C., Hornig-Do H. T., Bruni F., Chang J. H., Jourdain A. A., Martinou J. C., Falkenberg M., Spåhr H., Larsson N. G., Lewis R. J., Hewitt L., Baslé A., Cross H. E., Tong L., Lebel R. R., et al (2014) A human mitochondrial poly(A) polymerase mutation reveals the complexities of post-transcriptional mitochondrial gene expression. Hum. Mol. Genet. 23, 6345–6355 25008111 10.1093/hmg/ddu352 PMC4222368

[30] Barrientos A. (2015) Mitochondriolus: assembling mitoribosomes. Oncotarget 6, 16800–16801 26119155 10.18632/oncotarget.4646 PMC4627261

[31] Arroyo J. D., Jourdain A. A., Calvo S. E., Ballarano C. A., Doench J. G., Root D. E., and Mootha V. K. (2016) A Genome-wide CRISPR death screen identifies genes essential for oxidative phosphorylation. Cell Metab. 24, 875–885 27667664 10.1016/j.cmet.2016.08.017 PMC5474757

[32] Greber B. J., Boehringer D., Leitner A., Bieri P., Voigts-Hoffmann F., Erzberger J. P., Leibundgut M., Aebersold R., and Ban N. (2014) Architecture of the large subunit of the mammalian mitochondrial ribosome. Nature 505, 515–519 24362565 10.1038/nature12890

[33] Antonicka H., Choquet K., Lin Z. Y., Gingras A. C., Kleinman C. L., and Shoubridge E. A. (2017) A pseudouridine synthase module is essential for mitochondrial protein synthesis and cell viability. EMBO Rep. 18, 28–38 27974379 10.15252/embr.201643391 PMC5210091

[34] Hamma T., and Ferré-D'Amaré A. R. (2006) Pseudouridine synthases. Chem. Biol. 13, 1125–1135 17113994 10.1016/j.chembiol.2006.09.009

[35] Hoang C., Chen J., Vizthum C. A., Kandel J. M., Hamilton C. S., Mueller E. G., and Ferré-D'Amaré A. R. (2006) Crystal structure of pseudouridine synthase RluA: indirect sequence readout through protein-induced RNA structure. Mol. Cell 24, 535–545 17188032 10.1016/j.molcel.2006.09.017

[36] Dennerlein S., Rozanska A., Wydro M., Chrzanowska-Lightowlers Z. M., and Lightowlers R. N. (2010) Human ERAL1 is a mitochondrial RNA chaperone involved in the assembly of the 28S small mitochondrial ribosomal subunit. Biochem. J. 430, 551–558 20604745 10.1042/BJ20100757 PMC2995420

[37] Bakin A. V., and Ofengand J. (1998) Mapping of pseudouridine residues in RNA to nucleotide resolution. Methods Mol. Biol. 77, 297–309 9770678 10.1385/0-89603-397-X:297

[38] Powell C. A., Kopajtich R., D'Souza A. R., Rorbach J., Kremer L. S., Husain R. A., Dallabona C., Donnini C., Alston C. L., Griffin H., Pyle A., Chinnery P. F., Strom T. M., Meitinger T., Rodenburg R. J., et al (2015) TRMT5 mutations cause a defect in post-transcriptional modification of mitochondrial tRNA associated with multiple respiratory-chain deficiencies. Am. J. Hum. Genet. 97, 319–328 26189817 10.1016/j.ajhg.2015.06.011 PMC4573257

[39] Diodato D., Melchionda L., Haack T. B., Dallabona C., Baruffini E., Donnini C., Granata T., Ragona F., Balestri P., Margollicci M., Lamantea E., Nasca A., Powell C. A., Minczuk M., Strom T. M., et al (2014) VARS2 and TARS2 mutations in patients with mitochondrial encephalomyopathies. Hum. Mutat. 35, 983–989 24827421 10.1002/humu.22590 PMC4140549

