# Supplementary material for: The Pseudouridine Synthase RPUSD4 Is an Essential Component of Mitochondrial RNA Granules
Source: J Biol Chem. 2017 Jan 12;292(11):4519–32. doi: 10.1074/jbc.M116.771105 (PMC5377769; doi:10.1074/jbc.M116.771105)
Supplement: Supplemental Data [file supp_292_11_4519__index.html]

The Pseudouridine Synthase RPUSD4 Is an Essential Component of Mitochondrial RNA Granules — RPUSD4 Role in Mitochondrial Translation — Supplemental Data 

# The Pseudouridine Synthase RPUSD4 Is an Essential Component of Mitochondrial RNA Granules

## Supplemental Data

- Supplemental Data
