# Supplementary material for: The Pseudouridine Synthase RPUSD4 Is an Essential Component of Mitochondrial RNA Granules
Source: J Biol Chem. 2017 Jan 12;292(11):4519–32. doi: 10.1074/jbc.M116.771105 (PMC5377769; doi:10.1074/jbc.M116.771105)
Supplement: Supplemental Data [file supp_M116.771105_suppdata.pdf]

SUPPLEMENTARY INFORMATION

The pseudouridine synthase RPUSD4 is an essential component of mitochondrial RNA granules

Sofia Zaganelli[1], Pedro Rebelo-Guiomar[2,3], Kinsey Maundrell[1], Agata Rozanska[4], Sandra Pierredon[1], Christopher A. Powell[2], Alexis A. Jourdain[1,5], Nicolas Hulo[6], Robert N. Lightowlers[4], Zofia M. Chrzanowska-Lightowlers [4], Michal Minczuk[2], Jean-Claude Martinou[1]

Table of content:

Figure S1. Potential mitochondrial RNA binding proteins

Figure S2. Co-immunoprecipitation experiments of MRG-associated proteins

Figure S3. CRISPR/Cas9-derived RPUSD4 heterozygous cells show defective mitochondrial respiration

Table S1: Mass spectrometry results of co-immunoprecipitation experiments

Table S2: List of primers

RNA-interactome and Mitocarta 2.0 candidates

ABCF2	**ERAL1**	MGST3	MRPS2	**PUSL1**
ACAA2	ETFB	MMAB	MRPS21	RAB35
ACLY	FAM195A	***MRM1***	MRPS23	RARS
AK2	FASN	MRP63	MRPS24	***RNMTL1***
AKAP1	***FASTK***	MRPL1	MRPS25	RPL10A
ALAS2	**FASTKD1**	MRPL11	MRPS27	RPL34
ALDH181	***FASTKD2***	MRPL13	MRPS28	RPL35A
ALDH1L2	FASTKD3	MRPL14	MRPS30	RPS14
ALDH6A1	***FASTKD5***	MRPL15	MRPS31	RPS15A
ALDH7A1	FDPS	MRPL17	MRPS35	**RPUSD3**
ATP5A1	FKBP10	MRPL19	MRPS5	**RPUSD4**
ATP5B	FTH1	MRPL2	MRPS7	SARS
ATP5C1	***FTSJ2***	MRPL21	MRPS9	SDHA
ATP5I	GADD45GIP1	MRPL22	MRRF	SECISBP2
ATXN2	GAPDH	MRPL24	**MTERF2**	SHMT2
C14orf156	GARS	MRPL27	MTERFD1	SLC25A5
C17orf42	GATM	MRPL28	MTHFD1	**SND1**
C1orf31	**GFM1**	MRPL3	MTIF2	SSBP1
**C4orf14**	***GRSF1***	MRPL32	*MTPAP*	SUCLG1
C7orf30	GTPBP10	MRPL33	NDUFS6	***SUPV3L1***
CAT	HADH	MRPL34	NDUFV3	**TACO1**
**CHCHD3**	HADHB	MRPL37	NGRN	**TARS2**
**CHCHD7**	HARS2	MRPL38	OPA1	TBRG4
CISD1	HIBADH	MRPL39	P4HB	TFAM
CISD2	HRSP12	MRPL4	PACSIN2	**TFB1M**
COX4I1	HSDL2	MRPL40	**PARK7**	TFB2M
COX5B	HSPA9	MRPL41	PHB	TIMM44
CPS1	HSPD1	MRPL42	***PNPT1***	TKT
CYCS	HSPE1	MRPL43	***POLRMT***	TPI1
DAP3	IDH1	MRPL44	POR	TRAP1
DBI	IDH2	**MRPL45**	PPIF	TRMT1
DDX28	IMMT	MRPL48	PRDX2	TRMT61B
DECR1	KARS	MRPL49	PRDX3	**TRUB2**
DHCR24	***KIAA0391***	MRPL51	PRDX4	TSFM
DHX30	KIF1B	MRPL9	PRDX6	TUFM
DLD	LAMC1	MRPS10	PROSC	TXNRD2
DMGDH	LDHB	MRPS11	PSMA6	WBSCR16
DNAJA3	LONP1	MRPS14	**PTCD1**	XPNPEP3
DNM1L	LRPPRC	MRPS15	**PTCD2**	**YARS2**
DUT	MCAT	MRPS17	**PTCD3**	
ECH1	MDH1	MRPS18A	PTRH1	
ELAC2	MDH2	MRPS18B	**PUS1**	

Other candidates

**AFG3L2**	**MRPS7**	**mtIF3**	**PDE12**	**YMEL1**
**CH60**	**MRPS16**	**NSUN4**	**REXO2**	
**MRPL47**	**MRPS9**	**OXA1L**	**YBEY**	

Figure S1. Potential mitochondrial RNA binding proteins

Upper panel: All 207 RNA binding proteins present in Mitocarta 2.0. Lower panel: Other proteins in Mitocarta 2.0 not known to bind RNA but tested as potential components of MRGs. In bold are indicated the proteins that we further investigated for their localization, in italics are indicated the MRG-associated proteins already published.

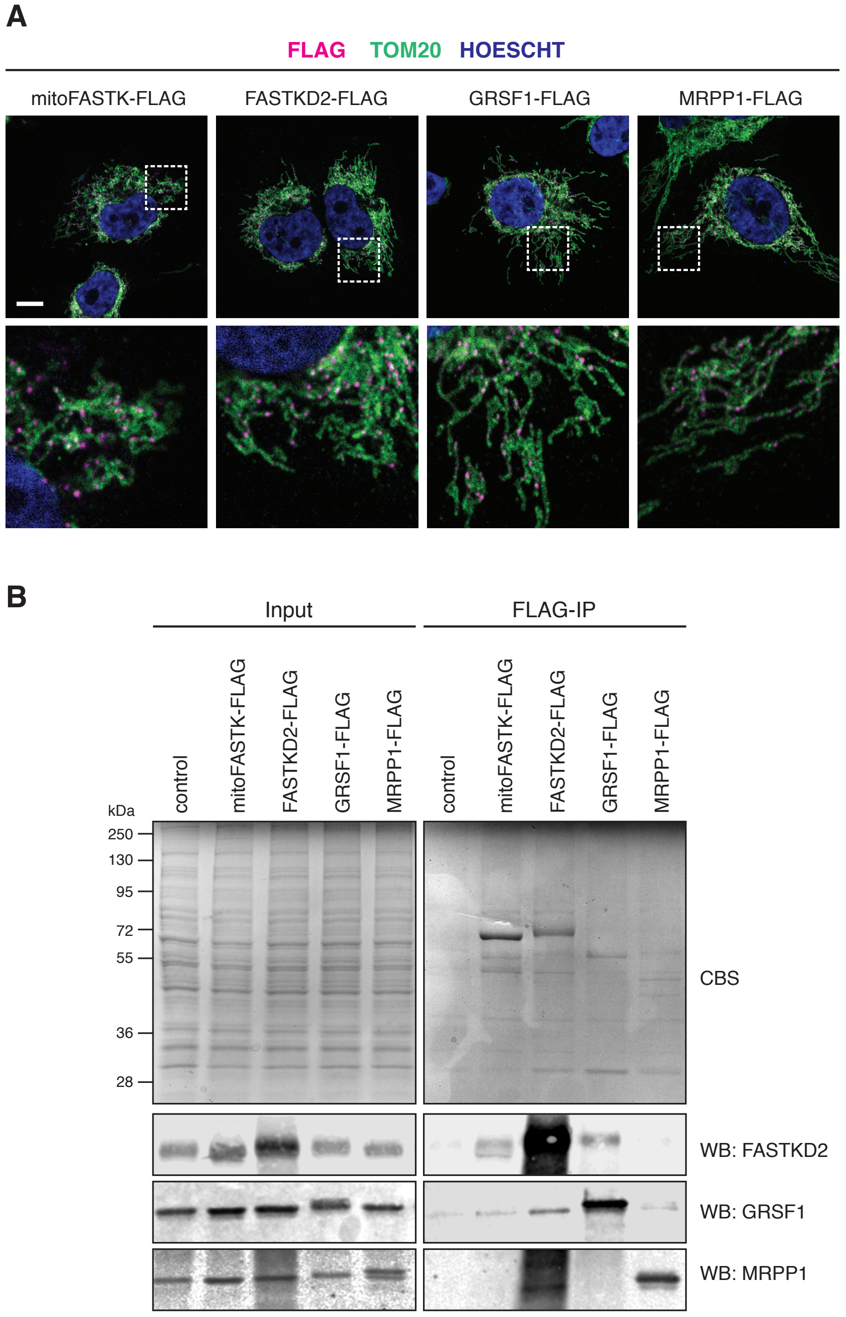

Figure S2. Co-immunoprecipitation experiments of MRG-associated proteins
(A) Confocal analysis to verify the expression and the proper localization of FLAG-tagged mitoFASTK, FASTKD2, GRSF1 and MRPP1. 143B cells stably expressing the indicated FLAG-tagged proteins were immunolabelled with anti-FLAG and anti-TOM20, as a mitochondrial marker. White boxes indicate the regions shown at higher magnification. Scale bar is 10 µm. (B) Coomassie gel staining and immunoblot analysis of immunoprecipitated proteins. To visualize proteins, the gels were stained using coomassie blue staining (CBS), and in parallel, to verify the correct co-immunoprecipitation of the indicated proteins, immunoblot analyses of FASTKD2, GRSF1 and MRPP1 were performed.

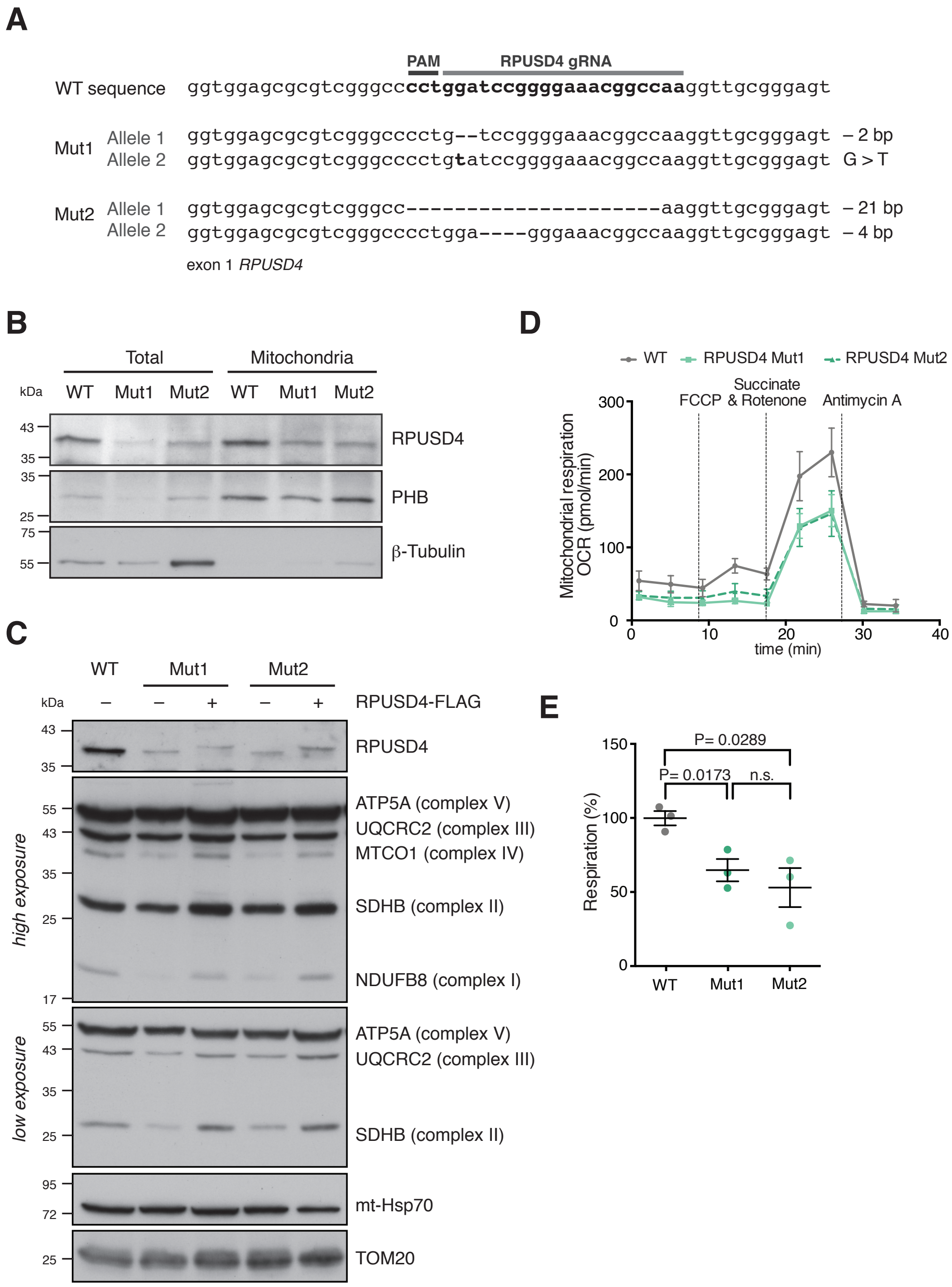

Figure S3. CRISPR/Cas9-derived RPUSD4 heterozygous cells show defective mitochondrial respiration

(A) Sequence analysis of *RPUSD4* exon 1 showing the mutations present in the 2 viable clones (Mut1 and Mut2) obtained following CRISPR/Cas9-mediated gene disruption. In both cases, the targeted regions in *RPUSD4* gene lie within the sequence encoding the mitochondrial targeting signal. (B) RPUSD4 mutations present in Mut1 or Mut2 do not impair mitochondrial import. Western blotting of mitochondrial fractions of the 2 mutant cell lines shows only ~50% depletion of the endogenous RPUSD4, consistent with their genotypes. The purity of the mitochondrial fractions was assessed using β-Tubulin and PHB as cytosolic and mitochondrial markers respectively. (C) SDS-PAGE analysis shows reduced steady-state levels of respiratory chain components in RPUSD4 Mut1 and Mut2 heterozygous cells. Western blot membranes were probed with an antibody cocktail specific for components of each of the OXPHOS complexes. mtHSP70 and TOM20 were used as loading controls. Due to differences in the signals for the respiratory chain subunits, high and low exposures of the blot are presented. (D) Partially reduced levels of RPUSD4 cause decreased mitochondrial respiration. Representative profiles of oxygen consumption rates (OCR) in the XF-PMP

permeabilized cell lines are indicated. Assays were performed in the presence of pyruvate (10 mM) and malate (1 mM) as carbon sources, and the following compounds were injected at the times indicated by vertical lines. FCCP, carbonyl cyanide-4-(trifluoromethoxy)phenylhydrazone (2 μM); succinate (10 mM) and rotenone (1 μM); antimycin A (1 μM). All measurements were made in the presence of oligomycin (1 μM) to inhibit ATP-linked respiration. Data are represented as means ± SD. (E) Oligomycin-sensitive respiration was normalized to antimycin A-driven respiration and expressed as % of respiration relative to wild-type (WT) cells. Data from 3 independent experiments are represented as means ± SEM. *P* values were obtained using the Student's t-test.

Table S1: Mass spectrometry results of co-immunoprecipitation experiments

	Mass spectrometry results of co-immunoprecipitation experiments	RELATIVE PEPTIDE ABUNDANCE					ENRICHEMENT OVER CONTROL (FOLD)				
**SYMBOL**	**GENE NAME**	**CONTROL**	**mitoFASTK**	**FASTKD2**	**GRSF1**	**MRPP1**	**mitoFASTK**	**FASTKD2**	**GRSF1**	**MRPP1**	**NUMBER OF IPs**
GRSF1	G-rich sequence factor 1	0	9,1642	7,9257	52,37	4,8591	∞	∞	∞	∞	4
L2HGDH	L-2-hydroxyglutarate dehydrogenase	0	8,0187	5,2838	3,4341	3,8873	∞	∞	∞	∞	4
PNPT1	Polyribonucleotide nucleotidyltransferase 1	0	5,7276	6,1645	2,5756	2,9155	∞	∞	∞	∞	4
FASTKD2	FAST kinase domain-containing protein 2	5,1888	14,892	126,81	23,18	7,7746	2,870027752	24,43917669	4,467314215		3
GCAT	2-amino-3-ketobutyrate coenzyme A ligase	0	5,7276	7,9257	3,4341	0	∞	∞	∞		3
GCDH	Glutaryl-CoA dehydrogenase	0	5,7276	2,6419	0	2,9155	∞	∞		∞	3
KIAA0391	Mitochondrial ribonuclease P protein 3	0	4,5821	4,4032	0	2,9155	∞	∞		∞	3
MRPL22	39S ribosomal protein L22	0	0	4,4032	3,4341	4,8591		∞	∞	∞	3
MRPS35	28S ribosomal protein S35	0	0	3,5226	3,4341	2,9155		∞	∞	∞	3
PDPR	Pyruvate dehydrogenase phosphatase regulatory subunit	0	8,0187	5,2838	0	3,8873	∞	∞		∞	3
STOML2	Stomatin-like protein 2	0	4,5821	5,2838	2,5756	0	∞	∞	∞		3
YARS2	Tyrosine--tRNA ligase	0	4,5821	7,9257	5,1511	0	∞	∞	∞		3
ATP5O	ATP synthase subunit	3,1133	9,1642	7,0451	6,0096	5,8309	2,943564706	2,262904314			2
BCS1L	Mitochondrial chaperone BCS1	0	0	3,5226	0	2,9155		∞		∞	2
CLPB	Caseinolytic peptidase B protein homolog	0	3,4366	2,6419	0	0	∞	∞			2
DHX30	Putative ATP-dependent RNA helicase DHX30	7,2643	30,929	15,851	12,878	11,662	4,257671076	2,18204094			2
FASTKD5	FAST kinase domain-containing protein 5	0	4,5821	6,1645	0	0	∞	∞			2
FOXRED1	FAD-dependent oxidoreductase domain-containing protein 1	0	3,4366	2,6419	0	0	∞	∞			2
GFM2	Ribosome-releasing factor 2	0	5,7276	2,6419	0	0	∞	∞			2
GRPEL2	GrpE protein homolog 2	0	0	0	2,5756	2,9155			∞	∞	2
HK2	Hexokinase-2	0	6,8731	3,5226	0	0	∞	∞			2
MRPL53	39S ribosomal protein L53	0	4,5821	2,6419	0	0	∞	∞			2
NDUFS8	NADH dehydrogenase [ubiquinone] iron-sulfur protein 8	0	5,7276	2,6419	0	0	∞	∞			2
NDUFV2	NADH dehydrogenase [ubiquinone] flavoprotein 2	0	0	0	2,5756	3,8873			∞	∞	2

NSUN4	5-methylcytosine rRNA methyltransferase NSUN4	0	0	3,5226	0	3,8873		∞		∞	2
PARS2	Probable proline--tRNA ligase	0	0	0	4,2926	3,8873			∞	∞	2
PDK1	[Pyruvate dehydrogenase (acetyl-transferring)] kinase isozyme 1	0	4,5821	4,4032	0	0	∞	∞			2
POLRMT	DNA-directed RNA polymerase	0	0	3,5226	0	3,8873		∞		∞	2
SLC25A22	Mitochondrial glutamate carrier 1	0	0	2,6419	2,5756	0		∞	∞		2
SLIRP	SRA stem-loop-interacting RNA-binding protein	0	5,7276	4,4032	0	0	∞	∞			2
SND1	Staphylococcal nuclease domain-containing protein 1	0	0	0	4,2926	5,8309			∞	∞	2
TIMM50	Mitochondrial import inner membrane translocase subunit TIM50	0	0	4,4032	3,4341	0		∞	∞		2
AFG3L2	AFG3-like protein 2	3,1133	0	8,8064	0	4,8591		2,828638422			1
AGK	Acylglycerol kinase	0	0	2,6419	0	0		∞			1
BDH1	D-beta-hydroxybutyrate dehydrogenase	0	0	2,6419	0	0		∞			1
C2orf47	Uncharacterized protein C2orf47	0	0	2,6419	0	0		∞			1
CPS1	Carbamoyl-phosphate synthase [ammonia]	0	0	2,6419	0	0		∞			1
DDX28	Probable ATP-dependent RNA helicase DDX28	0	0	5,2838	0	0		∞			1
DLAT	Dihydrolipoyllysine-residue acetyltransferase component of pyruvate dehydrogenase complex	0	3,4366	0	0	0	∞				1
DNAJA3	DnaJ homolog subfamily A member 3	5,1888	16,037	9,687	6,0096	6,8028	3,090695344				1
EARS2	Probable glutamate--tRNA ligase	0	3,4366	0	0	0	∞				1
ELAC2	Zinc phosphodiesterase ELAC protein 2	4,151	5,7276	3,5226	0	16,521				3,980004818	1
ERAL1	GTPase Era	3,1133	3,4366	7,0451	2,5756	4,8591		2,262904314			1
FASTK	Fas-activated serine/threonine kinase	0	57,276	0	0	0	∞				1
GFM1	Elongation factor G	8,302	17,183	15,851	6,8682	9,7182	2,069742231				1
HADHB	Trifunctional enzyme subunit beta	4,151	8,0187	5,2838	8,5852	5,8309			2,068224524		1
HSD17B10	3-hydroxyacyl-CoA dehydrogenase type-2	11,415	12,601	14,09	11,161	46,648				4,086552781	1
HSD17B4	Peroxisomal multifunctional enzyme type 2	0	0	0	0	2,9155				∞	1
HSDL2	Hydroxysteroid dehydrogenase-like protein 2	0	0	0	0	2,9155				∞	1
HSPD1	60 kDa heat shock protein	39,435	307	55,48	40,35	42,76	7,784962597				1
MPST	3-mercaptopyruvate sulfurtransferase	0	0	3,5226	0	0		∞			1
MRPL4	39S ribosomal protein L4	0	0	3,5226	0	0		∞			1

MRPS16	28S ribosomal protein S16	0	0	2,6419	0	0		∞			1
MRPS17	28S ribosomal protein S17	0	0	0	0	2,9155				∞	1
MRPS18A	28S ribosomal protein S18a	0	0	2,6419	0	0		∞			1
MRPS24	28S ribosomal protein S24	0	5,7276	0	0	0	∞				1
MRPS25	28S ribosomal protein S25	0	5,7276	0	0	0	∞				1
MRPS31	28S ribosomal protein S31	0	0	0	2,5756	0			∞		1
MTERFD1	mTERF domain-containing protein 1	0	0	4,4032	0	0		∞			1
MTERFD2	mTERF domain-containing protein 2	0	0	0	0	2,9155				∞	1
MTG2	Mitochondrial ribosome-associated GTPase 2	3,1133	8,0187	3,5226	0	0	2,575627148				1
MTHFD1	C-1-tetrahydrofolate synthase	0	0	0	3,4341	0			∞		1
NDUFAF7	NADH dehydrogenase [ubiquinone] complex I	4,151	3,4366	5,2838	5,1511	8,7464				2,10705854	1
NDUFB10	NADH dehydrogenase [ubiquinone] 1 beta subcomplex subunit 10	0	0	0	2,5756	0			∞		1
NDUFS2	NADH dehydrogenase [ubiquinone] iron-sulfur protein 2	4,151	9,1642	7,9257	2,5756	4,8591	2,207708986				1
OAT	Ornithine aminotransferase	6,2265	13,746	7,0451	6,8682	5,8309	2,207660805				1
OCIAD1	OCIA domain-containing protein 1	0	0	6,1645	0	0		∞			1
PRDX5	Peroxiredoxin-5	0	3,4366	0	0	0	∞				1
PTCD1	Pentatricopeptide repeat-containing protein 1	0	0	2,6419	0	0		∞			1
RARS2	Probable arginine--tRNA ligase	0	0	2,6419	0	0		∞			1
RPS14	40S ribosomal protein S14	0	0	0	3,4341	0			∞		1
RPUSD3	RNA pseudouridylate synthase domain-containing protein 3	0	0	3,5226	0	0		∞			1
RSAD1	Radical S-adenosyl methionine domain-containing protein 1	0	4,5821	0	0	0	∞				1
SLC16A1	Monocarboxylate transporter 1	0	0	4,4032	0	0		∞			1
SUCLG1	Succinyl-CoA ligase [ADP/GDP-forming] subunit alpha	0	0	2,6419	0	0		∞			1
TFB2M	Dimethyladenosine transferase 2	0	0	4,4032	0	0		∞			1
TOMM40	Mitochondrial import receptor subunit TOM40 homolog	0	0	3,5226	0	0		∞			1
TRMT10C	Mitochondrial ribonuclease P protein 1	3,1133	5,7276	6,1645	3,4341	63,169				20,29004593	1
TRNT1	CCA tRNA nucleotidyltransferase 1	0	0	0	0	3,8873				∞	1
TRUB2	Probable tRNA pseudouridine synthase 2	0	0	4,4032	0	0		∞			1

TST	Thiosulfate sulfurtransferase	4,151	11,455	7,0451	3,4341	3,8873	∞				1
WBSCR16	Williams-Beuren syndrome chromosomal region 16 protein	0	0	2,6419	0	0		∞			1
YME1L1	ATP-dependent zinc metalloprotease YME1L1	0	0	4,4032	0	0		∞			1

Table S2. List of primers

	FWD 5’-3’	REV 5’-3’
ND1	CTA CGC CCT GAT CGG CGC AC	CGG TAA TAC GAC TCA CTA TAG GGA GAG GTC GTA GCG GAA TCG GGG G
ND2	CAC CCC TCT GAC ATC CGG CCT	CGG TAA TAC GAC TCA CTA TAG GGA GAG GCC TCC TAG GGA GAG GAG GGT
ND3	CCA CCC CTT ACG AGT GCG GC	CGG TAA TAC GAC TCA CTA TAG GGA GAA GGC CAG ACT TAG GGC TAG GAT GA
ND5	ACG CCC GAG CAG ATG CCA AC	CGG TAA TAC GAC TCA CTA TAG GGA GAG GCG CAG ACT GCT GCG AAC A
CO1	TGG AGG CCG GAG CAG GAA CA	CGG TAA TAC GAC TCA CTA TAG GGA GAC GGC GGG GTC GAA GAA GGT G
CO2	CGC CCT CCC ATC CCT ACG CA	CGG TAA TAC GAC TCA CTA TAG GGA GAT ACC CCC GGT CGT GTA GCG G
ATP6/8	TGG CCC ACC ATA ATT ACC CCC A	CGG TAA TAC GAC TCA CTA TAG GGA GAG GGG GCA ATG AAT GAA GCG AAC AG
CYB	CTC ACT CCT TGG CGC CTG CC	CGG TAA TAC GAC TCA CTA TAG GGA GAG CCT CAC GGG AGG ACA TAG CC
12S	ACT CAA AGG ACC TGG CGG TGC	CGG TAA TAC GAC TCA CTA TAG GGA GAG GTG ACG GGC GGT GTG TAC G
16S	CCG TGA AGA GGC GGG CAT AAC AC	CGG TAA TAC GAC TCA CTA TAG GGA GAA TCC AAC ATC GAG GTC GTA AAC CCT
Met-tRNA	AGT AAG GTC AGC T	CGG TAA TAC GAC TCA CTA TAG GGA GAT AGT ACG GGA AGG
Val-tRNA	CAG AGT GTA GCT TAA CAC	CGG TAA TAC GAC TCA CTA TAG GGA GAT CAG AGC GGT CAA GTT AAG
Phe-tRNA	GTT TAT GTA GCT TAC CTC C	CGG TAA TAC GAC TCA CTA TAG GGA GAT GTT TAT GGG GTG ATG TGA G
7SL	GCC GGG CGC GGT GGC GCG TG	CGG TAA TAC GAC TCA CTA TAG GGA GAA GAG ACG GGG TCT CGC TAT G
5S	GTC TAC GGC CAT ACC ACC CTG	AAA GCC TAC AGC ACC CGG TAT
